# CsGATA5‐*CsTRYS/CsTDC2* module coordinates tryptophan‐mediated wound healing in postharvest citrus fruit

**DOI:** 10.1111/tpj.71170

**Published:** 2026-10-16

**Authors:** Xiaoquan Gao, Xiaojiao Li, Jialin Chen, Wenjun Wang, Ou Chen, Lanhua Yi, Kaifang Zeng

**Affiliations:** ^1^ College of Food Science Southwest University Chongqing 400715 People's Republic of China; ^2^ China‐Hungary Belt and Road Joint Laboratory on Food Science Southwest University Chongqing 400715 People's Republic of China; ^3^ College of Horticulture and Landscape Architecture Southwest University Chongqing 400715 People's Republic of China

**Keywords:** wound healing, citrus fruit, transcription factor, suberin biosynthesis, amino acid metabolism

## Abstract

The wound healing process in postharvest citrus fruit represents a crucial physiological mechanism that effectively resists external stress and minimizes quality deterioration. Nevertheless, the molecular mechanisms involved in wound healing by GATA transcription factors in citrus remain largely unexplored. In this research, CsGATA5 was characterized as a newly identified transcriptional activator promoting wound healing in citrus fruit. Transient overexpression of *CsGATA5* in tobacco also confirmed its role in promoting suberin accumulation. Gene expression and metabolite profiling analyses following *CsGATA5* overexpression revealed that CsGATA5 activated pathways associated with amino acid metabolism during wound healing. DLR, EMSA, ChIP‐qPCR, and Y1H assays demonstrated that CsGATA5 bound to the promoter regions of target genes in tryptophan metabolism (*CsTRYS* and *CsTDC2*), thereby activating their expression. Notably, the activation of *CsTRYS* and *CsTDC2* genes during citrus wound healing contributed to the subsequent induction of downstream suberin biosynthesis. Moreover, the exogenous treatment of key metabolites of the amino acid pathway, including tryptophan, 5‐hydroxytryptamine, and phenylalanine, significantly promoted wound healing and further supported a potential regulatory cascade of tryptophan metabolism– tricarboxylic acid cycle– suberin biosynthesis. This study sheds light on the molecular regulatory network by which GATA transcription factors modulate wound healing in fruit.

## Significance Statement

This study identifies CsGATA5 as a key transcription factor promoting wound healing in citrus fruit, activating a crucial tryptophan‐to‐suberin biosynthesis pathway. This reveals a novel regulatory module where tryptophan metabolism drives the synthesis of protective suberin to enhance fruit disease resistance.

## INTRODUCTION

Citrus fruit is particularly susceptible to mechanical injuries during harvesting, transportation, and storage, which may result in fungal infections and a reduction in fruit quality (Liao et al., 2025; Liu, Liu, et al., 2025). Scientific evidence indicates that postharvest citrus fruit exhibits an inherent wound healing ability that enables them to combat fungal pathogens and mitigate quality degradation, thus substantially minimizing storage‐related losses (Gao et al., 2024; Zhao et al., 2018). Moreover, considerable advancements have been achieved in researching citrus wound healing, primarily focusing on the key physiological pathways associated with the development of wound healing and the activation of these pathways by biotic and abiotic elicitors to enhance the healing process (Gao et al., 2024; Gao, Li, et al., 2025; Gao, Wang, et al., 2025; Huang et al., 2025; Li et al., 2026). For instance, research has identified that primary metabolic pathways related to citrus wound healing encompass amino acid and carbohydrate metabolic processes, while secondary metabolic pathways involve cell wall remodeling and suberin biosynthesis (Gao et al., 2024). Huang et al. (2025) demonstrated that *Debaryomyces nepalensis* can induce the accumulation of phenolic acid monomers, which are essential components of suberin, thus enhancing wound healing. Additionally, Gao, Li, et al. (2025) utilized key metabolites derived from carbohydrates to enhance glycometabolism, the tricarboxylic acid (TCA) cycle and energy‐related metabolic pathways, which support suberin accumulation and supply energy for tissue repair. Nevertheless, the exact molecular mechanisms that regulate these pathways during citrus wound healing remain to be fully elucidated.

Regulating crucial regulatory genes, especially transcription factors (TFs), involved in diverse developmental phases can enhance the efficiency of metabolite accumulation, rather than modifying a single structural gene (Han et al., 2023; Hernández‐García et al., 2024; Kim et al., 2024; Liu, Shi, et al., 2025; Wang, Chen, et al., 2023). Transcription factors are capable of recognizing specific DNA motifs to either induce or inhibit the transcription of target genes, thus modulating downstream metabolic pathways (Han et al., 2023; Hernández‐García et al., 2024). Wound healing is a crucial physiological response in plants when subjected to injury, playing a significant role in mitigating external stress and preventing disease onset (Lee et al., 2025; Wahrenburg et al., 2021). Suberin serves as an key compound in the wound healing process response of plants (Wahrenburg et al., 2021). Arabidopsis and potato, as established model organisms for studying this response, have been the focus of substantial research progress (Cohen et al., 2020; Gou et al., 2017; Han et al., 2024; Kosma et al., 2014; Lashbrooke et al., 2016; Verdaguer et al., 2016; Wen et al., 2025; Yang et al., 2025). A pivotal study elucidates the physiological role of AtMYB41, the first identified key transcription factor participate in regulating suberin biosynthesis (Kosma et al., 2014). Since this discovery, the roles of other TFs have been experimentally validated, demonstrating their close association with suberin biosynthesis in model organisms. For example, AtSUB and AtMYB107 are known to play roles in regulating suberin deposition in Arabidopsis (Cohen et al., 2020; Gou et al., 2017). StNAC103 is associated with suberin biosynthesis in potato (Verdaguer et al., 2016). AtATML1 and AtMYB74 are involved in the differentiation and specialization of epidermal cells during wound repair in *Arabidopsis thaliana* (Lee et al., 2025; Wen et al., 2025). In addition, AtMYB107 has been demonstrated to play a role in controlling suberin formation in conjunction with AtMYB9 in Arabidopsis (Lashbrooke et al., 2016). Notably, StWRKY20 and StMYB168 have been experimentally confirmed to interact and regulate phenolic component synthesis in potato suberin (Yang et al., 2025), whereas StMYB24 and StMYB144 are implicated in the lipid biosynthesis pathway of suberin in potato (Han et al., 2024). Moreover, accumulating evidence suggests that TFs are involved in modulating fruit wound healing. For instance, BTH was found to upregulate the transcriptional activity of MdMYC2, MdGAIPB, and MdMYB108 in apple (Deng et al., 2023). Several HuMYB, HubHLH, and HuAP2‐EREBP members were identified as potential regulators of damage‐stimulated the production of phenolic compounds in pitaya (Li et al., 2021). In kiwifruit, AcNAC2‐4, AcbZIP12, AcMYB70, AcABF1‐1, and AcMYC2 regulate crucial genes involved in the ethylene, abscisic acid, and jasmonic acid signaling pathways, collectively influencing the synthesis of ethylene and JA as well as suberin deposition during wound healing (Han et al., 2019; Han et al., 2022; Nieuwenhuizen et al., 2021; Wei et al., 2024; Wei, Wei, et al., 2022). PbMYB1 and CmWRKY1 have been recognized as critical components in modulating fatty acids and phenolic compounds of suberization in pear fruit and muskmelon fruit, respectively (Wang et al., 2022; Wang et al., 2024). Nevertheless, the molecular mechanisms through which transcription factors regulate distinct pathways linked to wound healing in citrus fruit remain largely underexplored.

GATA transcription factors are present across plant and other species. These proteins specifically bind to the evolutionarily conserved WGATAR sequence motif within the regulatory sequences of their target genes, where W represents T or A, and R stands for G or A (Schwechheimer et al., 2022). Current evidence concerning the role of GATA transcription factors involved in regulating plant growth, development, and responses to biotic as well as abiotic stresses has been documented (An et al., 2020; Behringer et al., 2014; Fang et al., 2025; Mou et al., 2025; Richter et al., 2013; Schwechheimer et al., 2022; Shao et al., 2024; Wu et al., 2025; Zhang et al., 2018). For instance, research has demonstrated that AtGATA3, discovered in Arabidopsis, plays a crucial role in modulating the formation of meristems during growth and development (An et al., 2020). SlGATA22 enhances cold tolerance and seed development in tomato through modulation of the CBF signaling pathway (Wu et al., 2025). BrGATA21 mitigates postharvest quality decline in cabbage by facilitating the removal of ROS (Mou et al., 2025). In apple, MdGATA15 participates in the response to elevated temperature stress via direct binding to the promoter regions of genes associated with anthocyanin biosynthesis, leading to transcriptional repression of these targets (Fang et al., 2025). While evidence has highlighted the beneficial role of CsGATA12 in conferring resistance to citrus bacterial canker (Fan et al., 2024), functional studies on other GATA family transcription factors in citrus remain scarce. In our previous research, the CsGATA5 transcription factor gene exhibited significantly increased expression levels during wound healing in citrus fruit based on transcriptome data, which suggested that CsGATA5 might serve as a key regulatory factor in wound healing. Nevertheless, the precise transcriptional regulatory processes by which CsGATA5 participates in wound healing have yet to be completely understood.

Amino acids are essential for modulating the nutritional value and quality attributes of fruits and vegetables by participating in diverse cellular physiological activities (Zeier, 2013). These include maintaining cellular acid–base homeostasis, regulating organ senescence, contributing to phytoalexin biosynthesis, and mediating reactions to diverse biotic and abiotic stressors, including pathogen infection and environmental adversities (Dong et al., 2024; Watanabe et al., 2013; Zeier, 2013). Suberin, a complex polymeric matrix composed of aliphatic and phenolic components, is ubiquitously present in higher plants and functions as a primary physical and chemical barrier during stress conditions. While several genes linked to suberin biosynthesis have been explored (Gao et al., 2024; Gao, Li, et al., 2025; Gao, Wang, et al., 2025), the regulatory mechanisms governing its synthesis and deposition are only beginning to be elucidated. Notably, the production of aromatic amino acids—tryptophan, phenylalanine, and tyrosine—is strongly associated with the shikimate pathway (Tohge et al., 2013), which not only supplies their carbon skeletons but also generates precursors for numerous critical secondary metabolites, including suberin, a hallmark compound in wound healing. Furthermore, essential genes linked to amino acid metabolism during wound healing in citrus were identified based on previous transcriptome data (Gao et al., 2024), particularly through the Cs*TRYS/*Cs*TDC2* encoding of tryptophan synthase and tryptophan decarboxylase. Tryptophan is synthesized in fruit tissues following the expression of tryptophan synthase and is partially converted into 5‐hydroxytryptamine via tryptophan decarboxylase activity (Kang et al., 2009). Accumulating evidence reveals that the tryptophan‐mediated 5‐hydroxytryptamine biosynthesis pathway contributes to stress defense responses in various plant species, including rice (Ishihara et al., 2008), pepper (Park et al., 2009), tomato (Tsunoda et al., 2021), apple (Lv et al., 2024), and grape (Ma et al., 2017). In addition, phenylalanine shares the upstream shikimate biosynthetic pathway with tryptophan (Tzin & Galili, 2010) and is actively involved in fruit wound healing processes (Ackah et al., 2022; Han et al., 2024; Wei, Wei, et al., 2022; Yang et al., 2025; Zheng et al., 2022). Together, these findings establish a theoretical foundation for investigating the potential roles of tryptophan, 5‐hydroxytryptamine and phenylalanine in wound healing. Therefore, this study selected three typical substrates—namely tryptophan, 5‐hydroxytryptamine, and phenylalanine—for exogenous application, aiming to evaluate the molecular function of *CsTRYS/CsTDC2* in mediating wound healing in citrus.

In light of the foregoing background, this study was designed with the following objectives: (i) to examine the functional correlation between CsGATA5 and wound healing in citrus, (ii) to decipher the precise molecular pathways by which CsGATA5‐*CsTRYS/CsTDC2* modulate wound healing, and (iii) to clarify the cascade regulatory network of the suberin biosynthesis mediated by tryptophan. The outcomes of this research contribute novel perspectives for expanding the regulatory network associated with CsGATA5 transcription factor in the field of fruit wound healing.

## RESULTS

### Increased *CsGATA5* expression during citrus fruit wound healing

To investigate the potential involvement of CsGATA5 in wound healing, its transcriptional expression at multiple time points throughout the healing period was analyzed. Quantitative PCR analysis revealed a significant increase in *CsGATA5* expression levels from 1 to 9 days during wound healing relative to 0 days, with a pronounced and continuous increase observed between 0 and 5 days (Figure 1A). These results indicate that CsGATA5 might be critically involved in wound healing.

**Figure 1:**
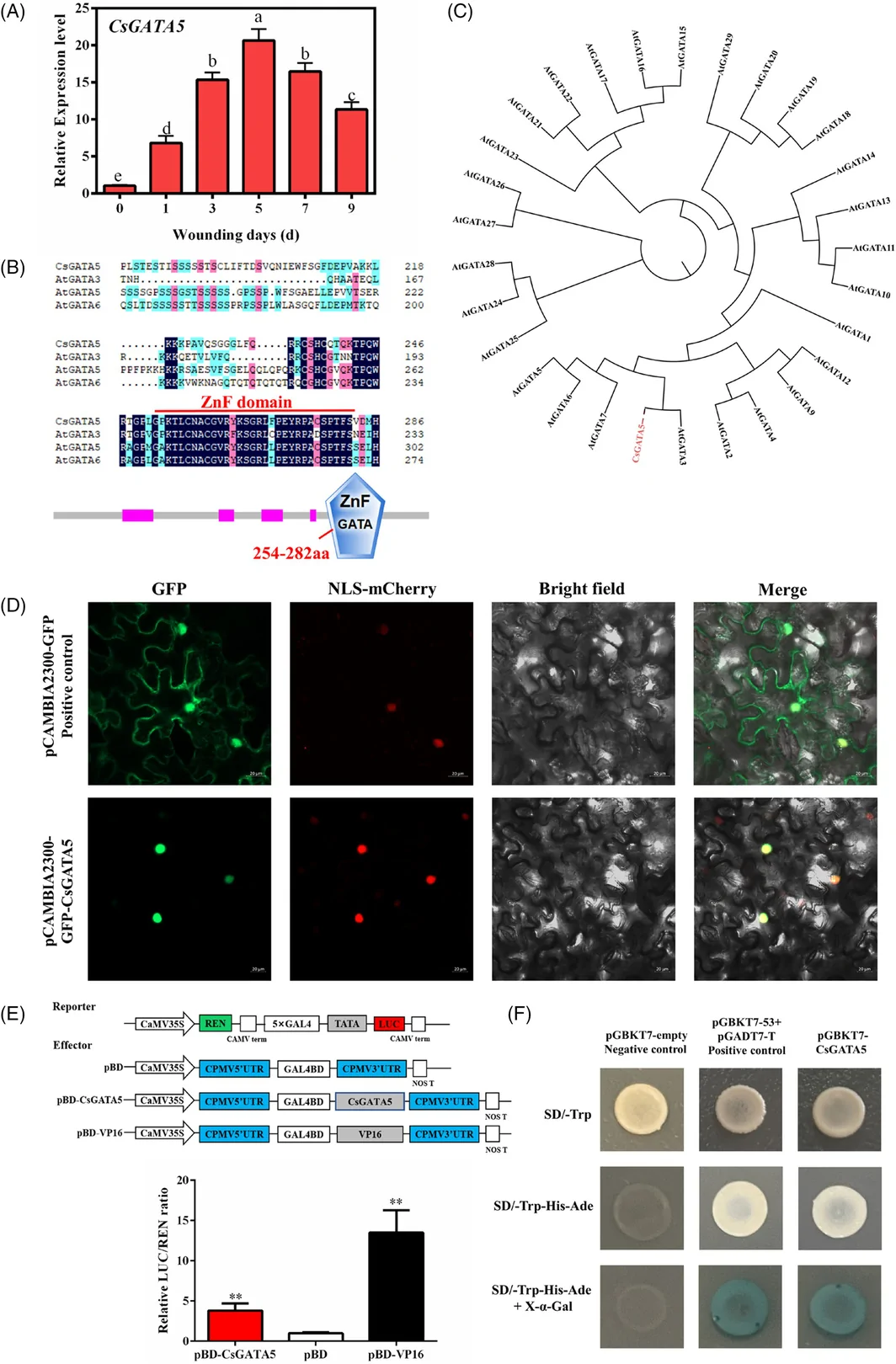
The expression profile and biological characteristics of CsGATA5. (A) The gene expression profile of CsGATA5 during the wound healing process in citrus fruit. (B) Multiple sequence alignment of the amino acid sequences of CsGATA5 with AtGATA3, AtGATA5, and AtGATA6. There was ZnF domain and labeled them using lines. (C) Phylogenetic tree constructed to illustrate the evolutionary relationship between CsGATA5 and Arabidopsis GATA transcription factors. (D) Subcellular localization analysis of CsGATA5. (E) Evaluation of the transcriptional activation capacity of CsGATA5 in *N. benthamiana* leaves. (F) Evaluation of the autoactivation potential of CsGATA5 in yeast. For (A), different letters denote significant differences (*P* < 0.05, one‐way ANOVA followed by Duncan's multiple‐range test); data are mean SD of three biological replicates (*n* = 3), each value averaged from three fruits. For (E), asterisks denote significant differences (**P < 0.01, Student's *t*‐test); Data are mean SD of three biological replicates (*n* = 3), each value averaged from three *N. benthamiana* leaves.

### Biological characteristics of CsGATA5

To gain deeper insights into biological characteristics of CsGATA5, amino acid sequence analysis and phylogenetic tree comparisons were performed. Sequence alignment revealed that CsGATA5 shared structural similarities with AtGATA3, AtGATA5, and AtGATA6 in *Arabidopsis thaliana* and contained a distinct zinc finger (ZnF) domain (Figure 1B), which suggested CsGATA5 demonstrated substantial DNA‐binding potential. Moreover, phylogenetic analysis confirmed a high degree of homology between CsGATA5 and the AtGATA3 transcription factor in *Arabidopsis thaliana* (Figure 1C). AtGATA3 has been demonstrated to be essential for regulating the meristem of Arabidopsis. This finding provided a potential clue that CsGATA5 might be involved in wound repair and the formation of healing tissue in citrus species.

To elucidate the fundamental molecular biological characteristics of the transcription factor CsGATA5, we performed subcellular localization analysis and transcriptional activation activity assays. The experimental results revealed that green fluorescence emitted by the GFP‐CsGATA5 fusion protein was exclusively localized within the nuclear compartment, whereas the control vector showed fluorescence diffusely present throughout both nuclear and membrane regions (Figure 1D). Subcellular localization analysis in tobacco revealed that CsGATA5 was localized in the nucleus. In comparison to the empty pBD vector, CsGATA5 exhibited a marked elevation in LUC/REN activity ratios in *N. benthamiana* leaves, consistent with the levels observed for the positive control pBD‐VP16 (Figure 1E). Furthermore, transcriptional autoactivation assays were conducted in yeast to analyze the transcriptional activation ability of CsGATA5. No cell growth was observed in the negative control group on SD/‐Trp‐His‐Ade selective medium; in contrast, cells transfected with the pGBKT7‐CsGATA5 plasmid as well as the positive control displayed robust proliferative activity under the same experimental conditions. Additionally, the inclusion of X‐α‐Gal led to the appearance of blue colonies (Figure 1F), which confirmed that CsGATA5 exhibited transcriptional activation activity in yeast cells. These findings collectively suggest that CsGATA5 functions as a transcriptional activator.

### CsGATA5 played a positive regulatory role in wound healing

Suberin deposition and disease resistance serve as critical indicators for assessing wound healing in citrus. To further explore the regulatory role of CsGATA5, we evaluated suberin accumulation and disease control in both citrus fruit and tobacco using overexpression systems. RT‐qPCR results verified a marked upregulation in gene expression levels following *CsGATA5* overexpression (*P* < 0.01), indicating the successful activation of the overexpression system in citrus (Figure 2A). Overexpression of *CsGATA5* markedly promoted suberin accumulation in citrus fruit (Figure 2B). In addition, the content of suberin monomer components in citrus significantly increased after overexpression of *CsGATA5* (Figure S1). *CsGATA5* overexpression significantly reduced disease incidence and lesion size in citrus relative to the control group on 3 days (Figure 2C–E). These findings demonstrated that *CsGATA5* overexpression facilitated wound healing of citrus fruit. To further confirm the biological function of CsGATA5 in this process, we conducted overexpression experiments in tobacco leaves and performed fluorescence imaging. Fluorescence observations revealed substantial suberin deposition on both the surface and transverse section of tobacco leaves following *CsGATA5* overexpression (Figure 2F; Figure S2). To further validate the regulatory role of CsGATA5 in wound healing, a virus‐induced gene silencing approach was employed to assess its impact on suberin deposition and the accumulation of suberin monomers. As illustrated in Figure S3A, the expression level of *CsGATA5* was markedly reduced following gene silencing, confirming the effectiveness of the silencing system in citrus. Silencing of *CsGATA5* inhibited the suberin biosynthesis in citrus fruit (Figure S3B,C). These experimental results support the hypothesis that CsGATA5 might positively regulate wound healing.

**Figure 2:**
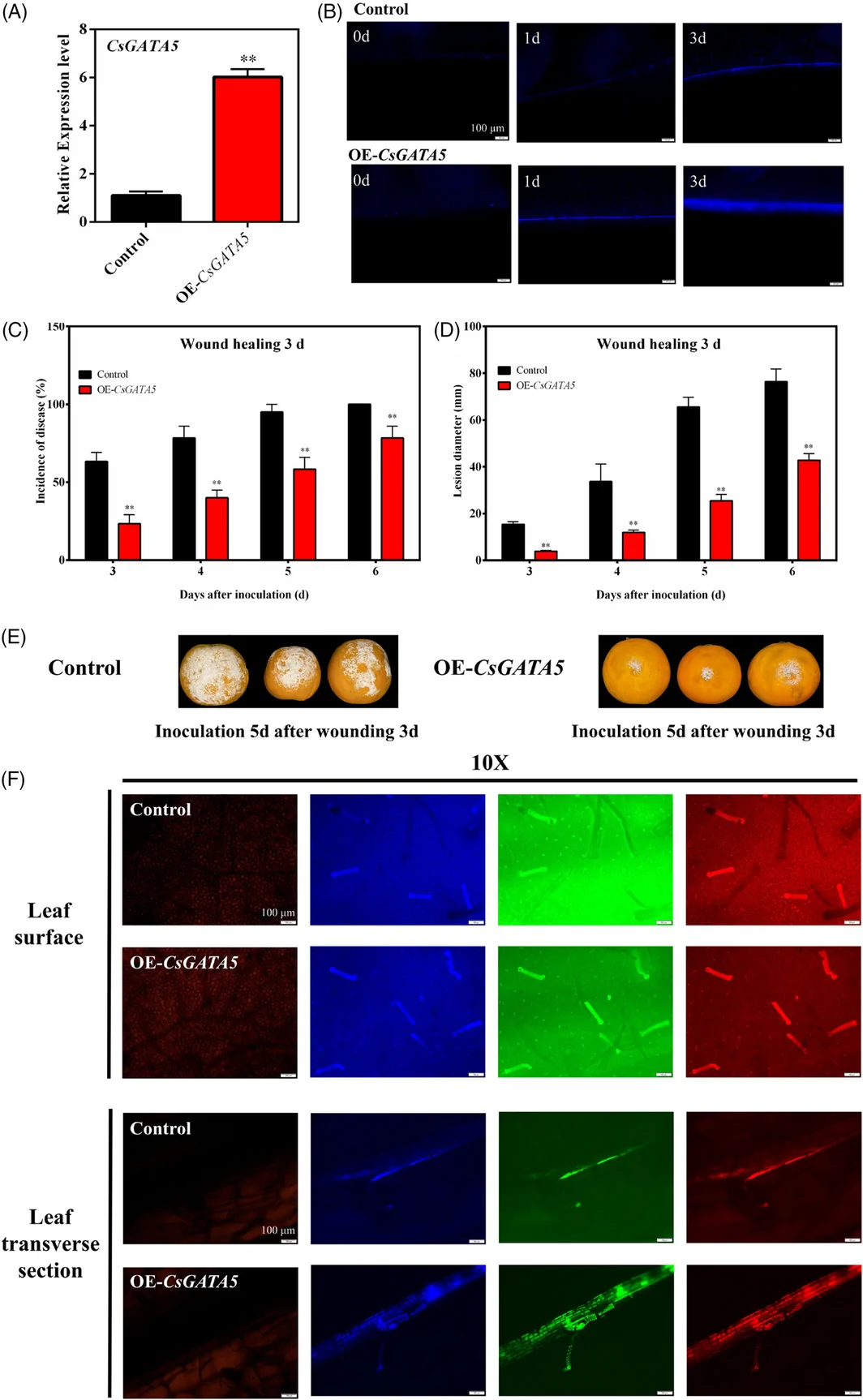
CsGATA5 played a positive regulatory role in wound healing. (A) RT‐qPCR was conducted to assess the expression levels of *CsGATA5* in citrus fruit following its overexpression. (B) Suberin deposition was examined using fluorescence imaging after *CsGATA5* overexpression in citrus fruit. (C) Incidence of disease was recorded subsequent to *CsGATA5* overexpression. (D) Lesion diameter was measured after overexpression of *CsGATA5*. (E) Morphological changes in citrus fruit following *CsGATA5* overexpression were visually evaluated and documented photographically 5‐day post‐inoculation. (F) Fluorescence of suberin accumulation was observed in *N. benthamiana* leaves following *CsGATA5* overexpression on the third day under 10 × magnification. For (A), (C), and (D), asterisks denote significant differences (**P < 0.01, Student's *t*‐test); data are mean SD of three biological replicates (*n* = 3), each value averaged from three fruits.

### CsGATA5 activated pathways associated with amino acid metabolism

A total of 4 amino acid metabolism genes have been identified as key regulators in citrus wound healing (Table S1). To further investigate the specific molecular mechanisms through which CsGATA5 participated in wound healing, the expression levels of key genes and the buildup of associated metabolites in amino acid metabolism following *CsGATA5* overexpression were examined. Compared with the control group, genes involved in amino acid metabolism, including *CsTRYS*, *CsTDC2*, and *CsCYS‐S1* (Figure 3A). Furthermore, the levels of several amino acids, specifically trp, cys, leu, met, phe, and tyr, were significantly elevated following *CsGATA5* overexpression (Figure 3B). The integrated examination of the upregulated key genes and corresponding metabolite levels suggested that CsGATA5 actively regulated the pathways of amino acid metabolism.

**Figure 3:**
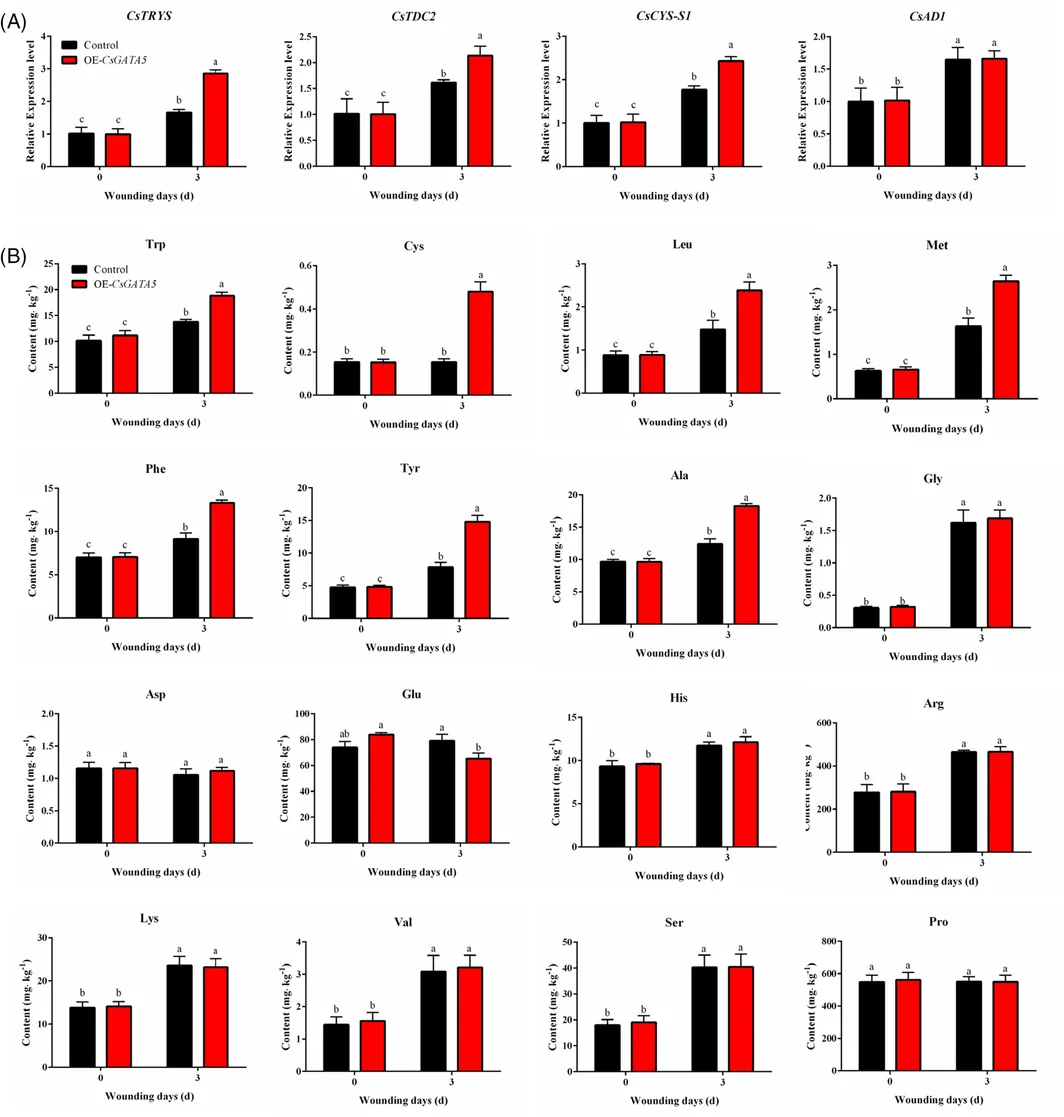
CsGATA5 activated amino acid metabolism in wound healing. (A) RT‐qPCR was conducted to assess the expression levels of amino acid metabolism genes following CsGATA5 overexpression. (B) Amino acid contents were quantified following *CsGATA5* overexpression. For (A) and (B), different letters denote significant differences (*P* < 0.05, one‐way ANOVA followed by Duncan's multiple‐range test); data are mean SD of three biological replicates (*n* = 3), each value averaged from three fruits.

### CsGATA5 promoted the transcriptional activation of *CsTRYS* and *CsTDC2* through direct binding to their promoters

Building upon the set of genes upregulated following *CsGATA5* overexpression, we conducted a further examination of cis‐regulatory elements located in the promoters of these genes. Among these upregulated genes, the promoters of *CsTRYS* and *CsTDC2* genes contain cis‐regulatory motifs, which might be recognized by CsGATA5 (Figure 4A). Specific target genes of CsGATA5 were subsequently identified through the application of dual‐luciferase reporter (DLR) assays, electrophoretic mobility shift assays (EMSA), Chromatin immunoprecipitation‐quantitative PCR (ChIP‐qPCR), and yeast one‐hybrid (Y1H) experiments. DLR analysis revealed a significant increase in the LUC/REN ratios of CsGATA5 upon co‐expression with pro‐LUC constructs of *CsTRYS* and *CsTDC2*, relative to the control (Figure 4B). However, mutation of the WGATAR motif to TTTTTT in the promoters of *CsTRYS* and *CsTDC2* abolished the transcriptional activation mediated by CsGATA5, as evidenced by the absence of a statistically significant difference in LUC/REN ratios relative to the control (Figure 4B). This finding suggests that CsGATA5 enhanced the promoter activity of *CsTRYS* and *CsTDC2*. Figure 4C,D illustrated that the EMSA assay was employed to further examine the binding capability of CsGATA5 to elements within *CsTRYS* and *CsTDC2* promoters *in vitro*. A distinct binding band was detected upon incubation of pCold‐His‐CsGATA5 with biotinylated probes derived from the promoters of the two genes. Furthermore, increasing the concentration of unlabeled probes markedly decreased the intensity of this band. Nevertheless, when the WGATAR motif in the mutant probe was substituted with the sequence TTTTTT, the specific DNA–protein binding bands corresponding to CsGATA5 interaction with the *CsTRYS* and *CsTDC2* promoter probes were abolished. These results indicated that CsGATA5 could interact with the promoter regions of *CsTRYS* (Figure 4C) and *CsTDC2* (Figure 4D) *in vitro*. Additionally, ChIP‐qPCR analysis further confirmed that CsGATA5 binds to these target genes. Anti‐CsGATA5 specifically precipitated promoter fragments of *CsTRYS* (Figure 4E) and *CsTDC2* (Figure 4F), respectively, offering direct evidence of CsGATA5's binding capability *in vivo* in citrus. In comparison to the control group, CsGATA5 exhibited enhanced growth on SD/‐His/‐Leu/‐Trp medium supplemented with a specific concentration of 3‐AT when co‐expressed with respective promoter constructs in Y1H assay. This result further demonstrated that CsGATA5 binds to the promoter regions of *CsTRYS* (Figure 4G) and *CsTDC2* (Figure 4H) *in vivo* in yeast.

**Figure 4:**
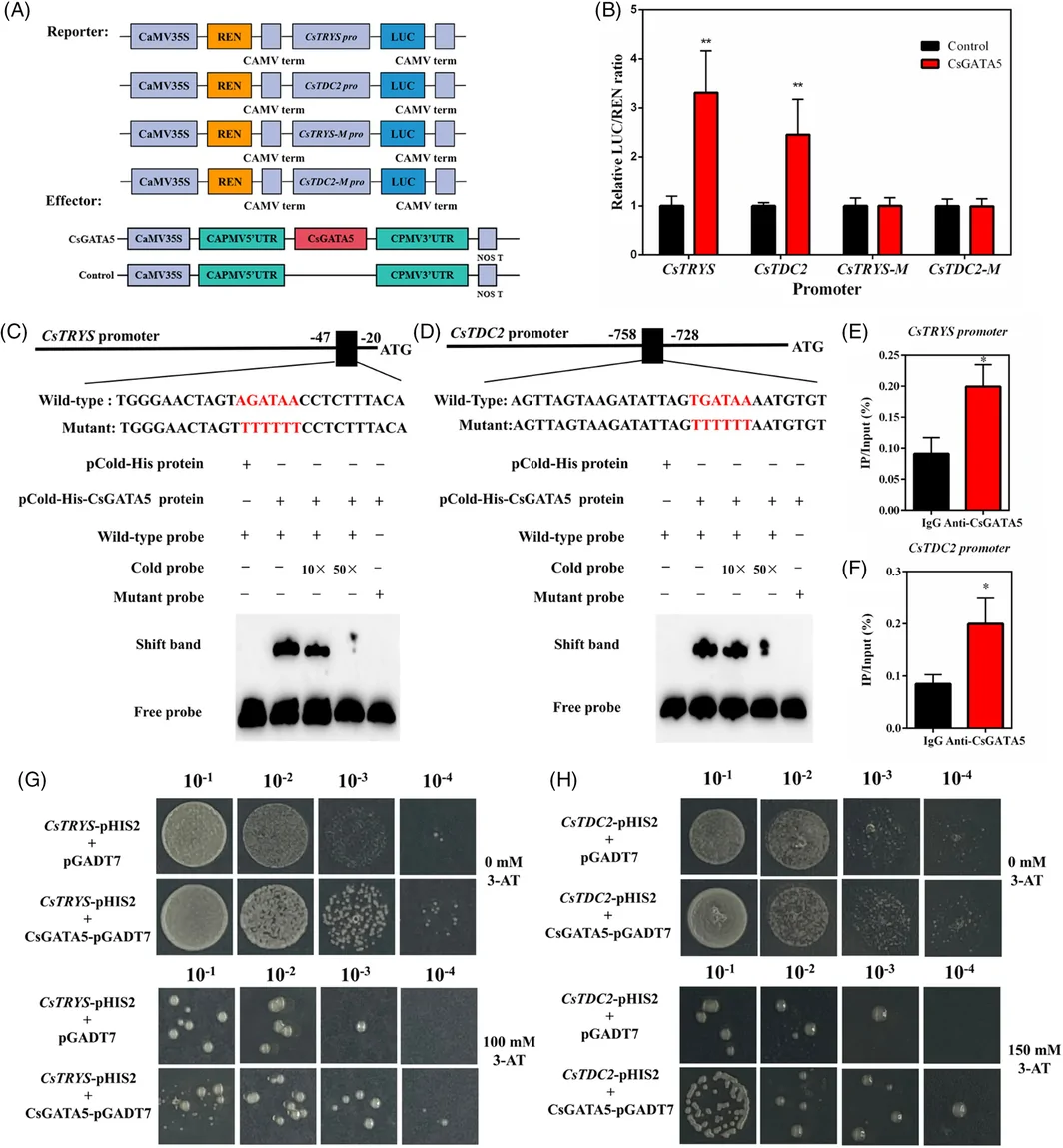
CsGATA5 promoted the transcriptional activation of *CsTRYS* and *CsTDC2* through direct binding to their respective promoter regions. (A) Diagrams depicting the construction of effector and reporter vectors employed in the DLR assay. (B) DLR assay implied that CsGATA5 activated the expression of *CsTRYS* and *CsTDC2*; *CsTRYS* and *CsTDC2* denote the wild‐type promoters harboring the WGATAR cis‐acting element, whereas *CsTRYS‐M* and *CsTDC2‐M* designate the corresponding mutant promoters in which the WGATAR motif has been substituted with a TTTTTTT sequence. (C) EMSA assay indicated CsGATA5 bound to *CsTRYS* promoter *in vitro*; The sequences of the WT probes containing the WGATAR motif were labeled with biotin. Competition for CsGATA5 binding was performed with 10× and 50× cold probes containing the cis‐element (WGATAR) or mutated control (TTTTTT). The symbols – and + represent absence and presence, respectively. (D) EMSA assay indicated CsGATA5 bound to *CsTDC2* promoter *in vitro*; The sequences of the WT probes containing the WGATAR motif were labeled with biotin. Competition for CsGATA5 binding was performed with 10× and 50× cold probes containing the cis‐element (WGATAR) or mutated control (TTTTTT). The symbols – and + represent absence and presence, respectively. (E) The binding of CsGATA5 to the promoters of *CsTRYS in vivo* confirmed by ChIP‐qPCR. (F) The binding of CsGATA5 to the promoters of *CsTDC2 in vivo* confirmed by ChIP‐qPCR. (G) Y1H assay demonstrated CsGATA5 bound to *CsTRYS* promoter *in vivo*. (H) Y1H assay demonstrated CsGATA5 bound to *CsTDC2* promoter *in vivo*. For (B), (E), and (F), asterisks denote significant differences (*P < 0.05, **P < 0.01, Student's *t*‐test); data are mean SD of three biological replicates (*n* = 3).

### Target genes *CsTRYS* and *CsTDC2* positively regulated wound healing

To investigate the connection between tryptophan metabolism and wound healing, particularly its association with suberin biosynthesis, we further investigated the effects of *CsTRYS* and *CsTDC2* overexpression on suberin deposition and disease resistance. As shown in Figure 5A,B, the transcript levels of *CsTRYS* and *CsTDC2* were markedly upregulated following overexpression in citrus. Overexpression of *CsTRYS* and *CsTDC2* markedly enhanced suberin deposition (Figure 5C). Notably, *CsTRYS* and *CsTDC2* are key genes implicated in tryptophan biosynthetic pathways. The considerable reduction in suberin components upon their suppression implies a potential regulatory connection between tryptophan metabolism and the biosynthesis of suberin in citrus. Moreover, overexpression of *CsTRYS* and *CsTDC2* significantly controlled disease in citrus fruit (Figure 5D–F). The expression levels of *CsTRYS* and *CsTDC2* were significantly downregulated after gene silencing (Figure S5A,B). Consistent with the control group, silencing *CsTRYS* and *CsTDC2* resulted in no significant suberin accumulation (Figure S5C). This finding further supports a strong correlation between tryptophan metabolism and suberin biosynthesis. These results indicate that the tryptophan metabolism genes *CsTRYS* and *CsTDC2* played a positive regulatory role in wound healing processes in citrus.

**Figure 5:**
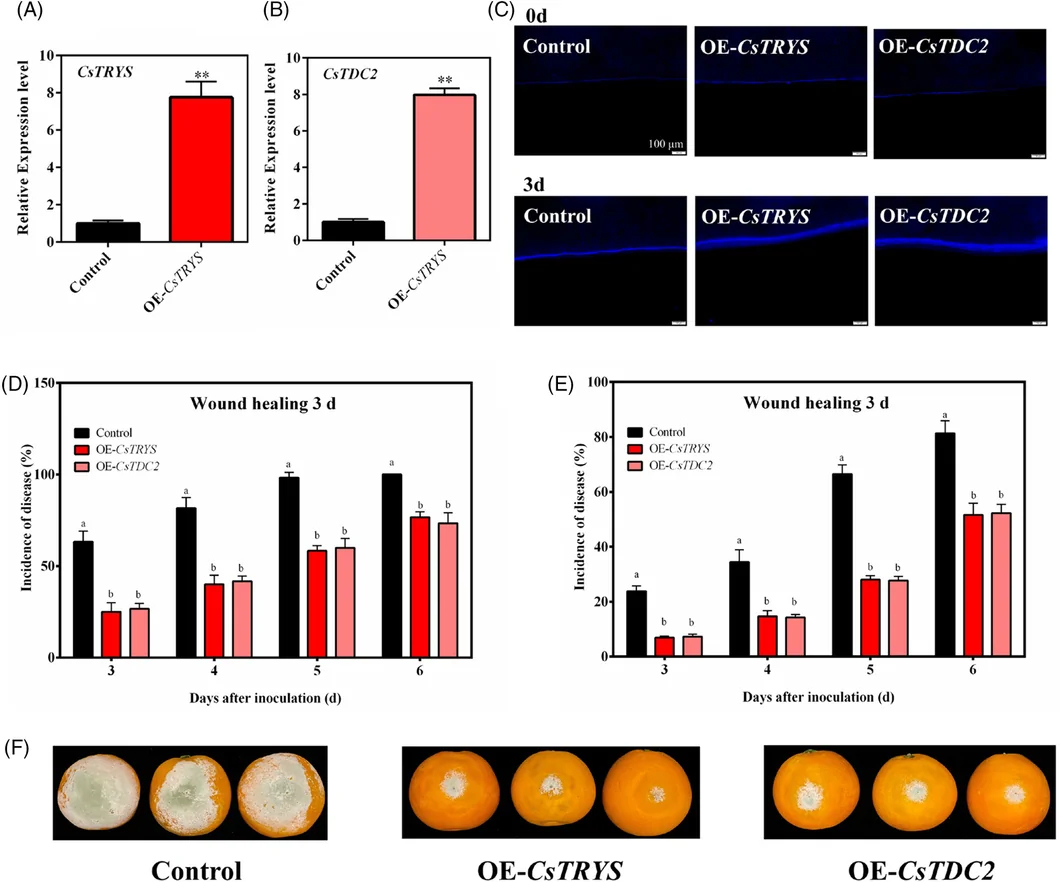
Target genes *CsTRYS* and *CsTDC2* positively regulated wound healing. (A) RT‐qPCR was conducted to assess the expression levels of *CsTRYS* in citrus fruit following its overexpression. (B) RT‐qPCR was conducted to assess the expression levels of *CsTDC2* in citrus fruit following its overexpression. (C) Suberin deposition was examined using fluorescence imaging after overexpression of *CsTRYS* and *CsTDC2* in citrus fruit. (D) Incidence of disease was measured after overexpression of *CsTRYS* and *CsTDC2*. (E) Lesion diameter was measured after overexpression of *CsTRYS* and *CsTDC2*. (F) Morphological changes in citrus fruit after overexpression of *CsTRYS* and *CsTDC2* were visually evaluated and documented photographically 5‐day post‐inoculation. For (A) and (B), asterisks denote significant differences (**P < 0.01, Student's *t*‐test); data are mean SD of three biological replicates (*n* = 3), each value averaged from three fruits. For (D) and (E), different letters denote significant differences (*P* < 0.05, one‐way ANOVA followed by Duncan's multiple‐range test); Data are mean SD of three biological replicates (*n* = 3), each value averaged from three fruits.

### Target genes *CsTRYS* and *CsTDC2* activated the downstream suberin biosynthesis through amino acid metabolism and tricarboxylic acid cycle

To further investigate the potential link between tryptophan metabolism and suberin biosynthesis, we subsequently assessed the effects of overexpressing the tryptophan metabolism‐associated genes *CsTRYS* and *CsTDC2* on amino acid content in citrus. The results revealed elevated levels of tryptophan, phenylalanine, and tyrosine following overexpression of *CsTRYS* and *CsTDC2* (Figure 6A). These amino acids were selected because they share a common upstream shikimate biosynthetic pathway and compete intensely for the pivotal intermediate, chorismate. Overexpression of tryptophan synthase and tryptophan decarboxylase substantially altered tryptophan‐related metabolism, which was accompanied by changes in phenylalanine and tyrosine pools, possibly through perturbation of shikimate pathway‐derived metabolic networks. As glucogenic and ketogenic amino acids, these metabolites are closely connected to TCA cycle‐associated metabolism. Moreover, overexpression of *CsTRYS* and *CsTDC2* resulted in elevated levels of key intermediates in carbohydrate metabolism and the TCA cycle, including fructose, glucose, deoxyglucose, pyruvate, acetyl‐CoA, and citric acid (Figure 6B). Furthermore, as a transcriptional regulatory factor upstream of *CsTRYS* and *CsTDC2*, overexpression of CsGATA5 significantly increased the levels of these core TCA intermediates at the wound site (Figure S4). This provides direct metabolic evidence that CsGATA5 drives tryptophan metabolism and further promotes the TCA cycle, which supplies both carbon skeletons and energy for suberin biosynthesis. Additionally, the levels of suberin monomer constituents were significantly elevated following overexpression of *CsTRYS* and *CsTDC2* (Figure 6C). These observations reveal a correlative link between enhanced amino acid and TCA cycle‐related metabolism and increased suberin monomer accumulation, implying that metabolic intermediates derived from amino acid metabolism and the TCA cycle may support downstream suberin biosynthesis. Furthermore, silencing of *CsTRYS* and *CsTDC2* led to a pronounced decline in suberin monomer contents (Figure S5D–F). Ultimately, these findings demonstrate that the tryptophan metabolism‐related genes *CsTRYS* and *CsTDC2* are important regulators governing suberin accumulation in citrus fruit.

**Figure 6:**
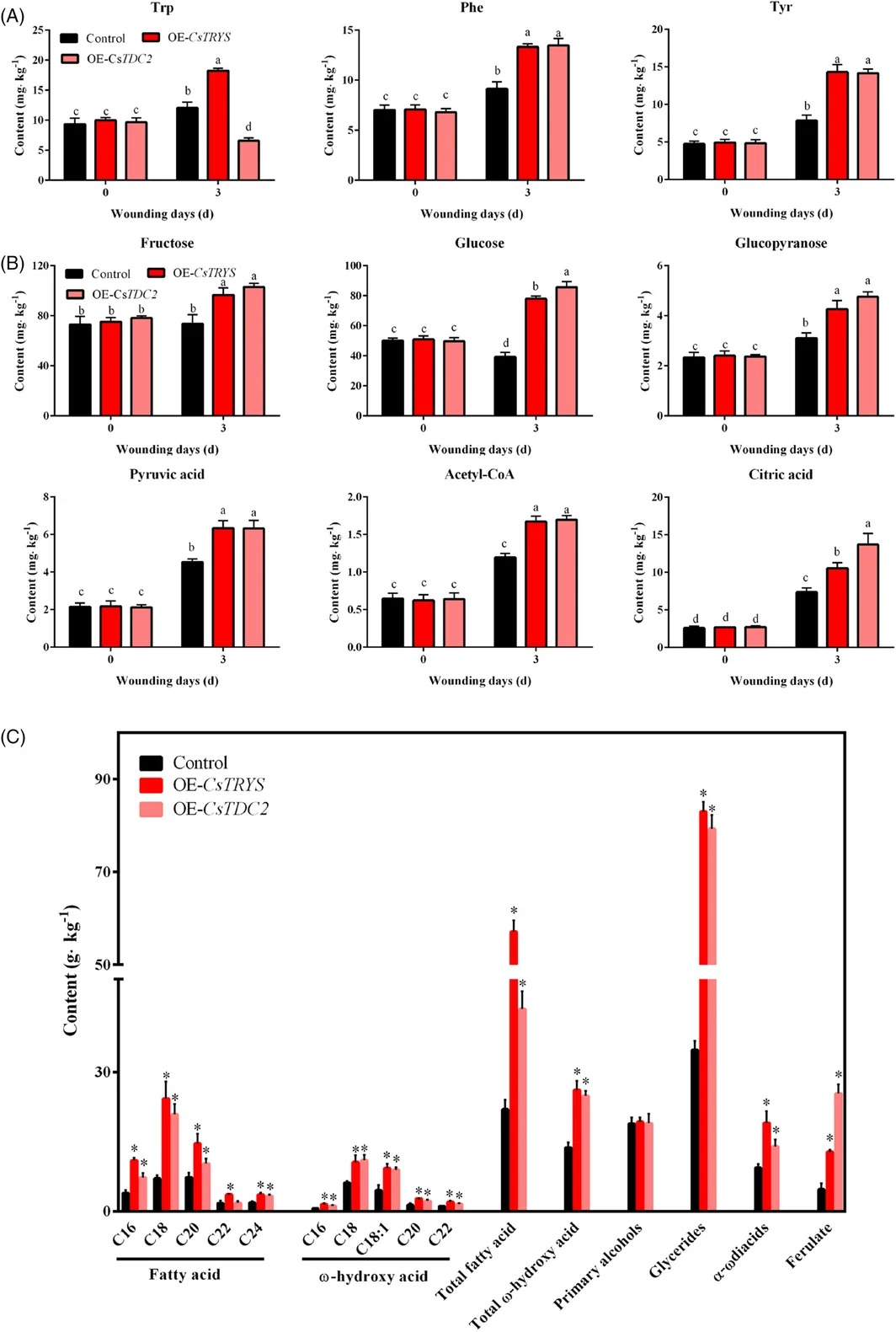
Overexpression of target genes *CsTRYS* and *CsTDC2* activated the downstream suberin biosynthesis pathway. (A) The contents of amino acids of citrus fruit were quantified following overexpression of *CsTRYS* and *CsTDC2*. (B) The contents of metabolites of citrus fruit in tricarboxylic acid (TCA) cycle were quantified after overexpression of *CsTRYS* and *CsTDC2*. (C) The contents of suberin monomer components of citrus fruit were quantified after overexpression of *CsTRYS* and *CsTDC2*. For (A) and (B), different letters denote significant differences (*P* < 0.05, one‐way ANOVA followed by Duncan's multiple‐range test); Data are mean SD of three biological replicates (*n* = 3), each value averaged from three fruits. For (C), asterisks denote significant differences (*P < 0.05, Student's *t*‐test); data are mean SD of three biological replicates (*n* = 3), each value averaged from three fruits.

### Exogenous tryptophan, 5‐hydroxytryptamine, and phenylalanine promoted wound healing by enhancing endogenous amino acid metabolism and stimulating the tricarboxylic acid cycle

To evaluate the potential role of tryptophan, 5‐hydroxytryptamine, and phenylalanine in involving wound healing, suberin deposition was examined in fruit tissues treated with exogenous applications of these compounds using fluorescence microscopy. As shown in Figure 7A, on 3 days of the healing process, significantly enhanced suberin deposition was observed in treated samples in comparison to the control group. To thoroughly evaluate the impacts of exogenous applications of tryptophan, 5‐hydroxytryptamine, and phenylalanine on wound healing, changes in disease incidence and lesion diameter following inoculation with *Penicillium digitatum* at various time points were also evaluated. Compared to the control, fruits treated with tryptophan, 5‐hydroxytryptamine, and phenylalanine showed significantly lower disease incidence and smaller lesion diameters after wound healing (Figure 7B–D). However, tryptophan, 5‐hydroxytryptamine, and phenylalanine did not significantly inhibit the growth of *P. digitatum* compared with the control group (Figure S6). The reduced fungal infection in citrus fruit is not attributed to direct antifungal activity, but rather to the suberization, which strengthens the physical barrier of citrus fruit against pathogen invasion. Collectively, these results support the hypothesis that tryptophan, 5‐hydroxytryptamine, and phenylalanine enhanced wound healing, thus decreasing susceptibility to postharvest diseases.

**Figure 7:**
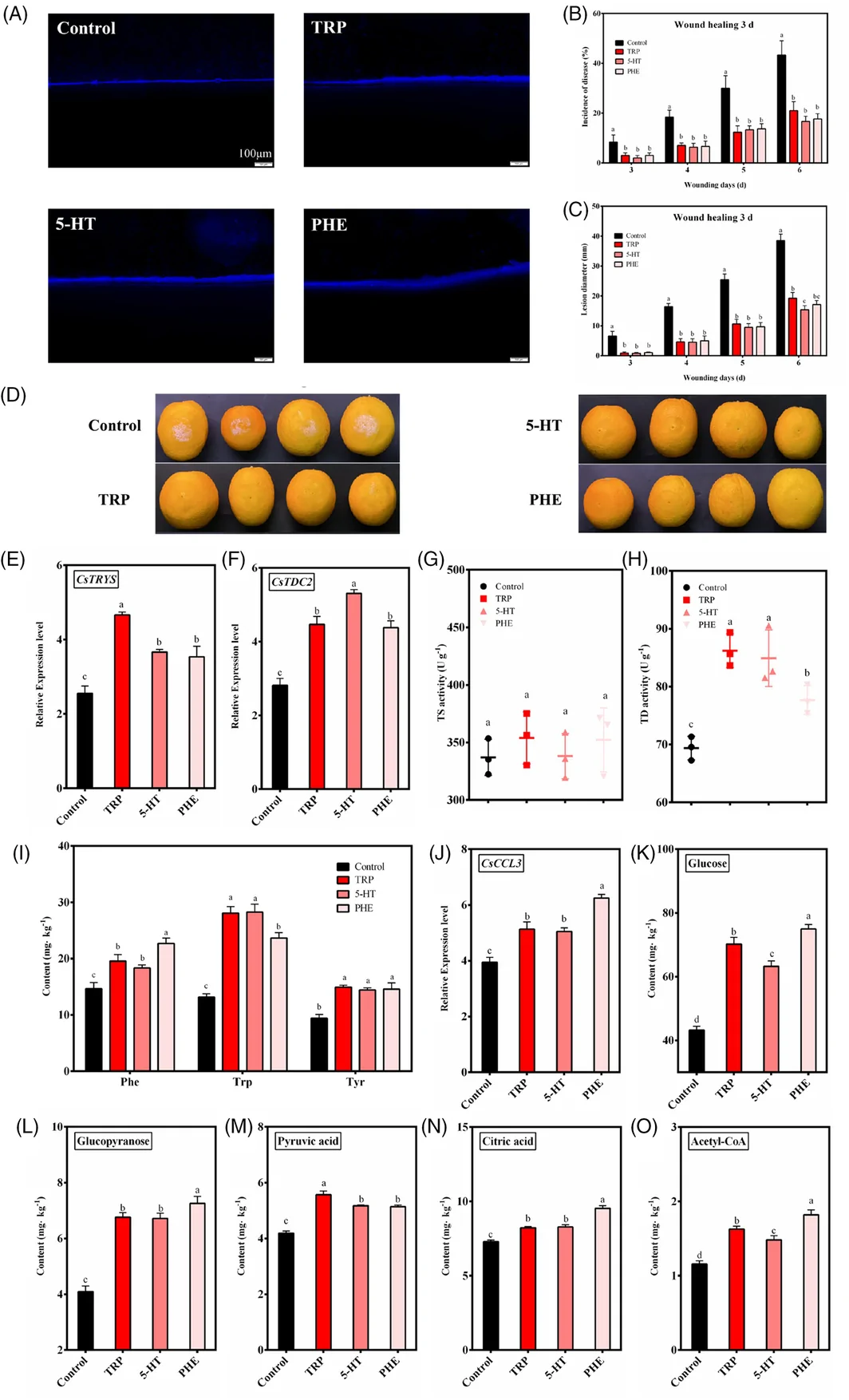
Exogenous tryptophan, 5‐hydroxytryptamine, and phenylalanine promoted wound healing by enhancing endogenous amino acid metabolism and stimulating the tricarboxylic acid cycle. (A) Fluorescence microscopic observation results of cuticle deposition at the wound site of citrus fruit treated with tryptophan, 5‐hydroxytryptamine, and phenylalanine on the third day of wound healing. (B) Effects of tryptophan, 5‐hydroxytryptamine, and phenylalanine on the incidence of citrus fruit diseases. (C) Effects of tryptophan, 5‐hydroxytryptamine, and phenylalanine on disease diameter on citrus fruit. (D) Morphological observation of citrus fruit treated with tryptophan, 5‐hydroxytryptamine, and phenylalanine after 3 days of healing and 6 days of inoculation with *Penicillium digitatum*. (E) Effects of tryptophan, 5‐hydroxytryptamine, and phenylalanine treatments on the expression level of *CsTRYS* involved in amino acid metabolism. (F) Effects of tryptophan, 5‐hydroxytryptamine, and phenylalanine treatments on the expression level of *CsTDC2* involved in amino acid metabolism. (G) Effects of tryptophan, 5‐hydroxytryptamine, and phenylalanine treatments on TS activity associated with amino acid metabolism. (H) Effects of tryptophan, 5‐hydroxytryptamine, and phenylalanine treatments on TD activity associated with amino acid metabolism. (I) Effects of tryptophan, 5‐hydroxytryptamine, and phenylalanine treatments on the endogenous levels of phenylalanine, tryptophan, and tyrosine in citrus fruit. (J) Effects of tryptophan, 5‐hydroxytryptamine, and phenylalanine treatments on the expression level of the *CsCCL3* gene involved in tricarboxylic acid cycle. (K) Effects of tryptophan, 5‐hydroxytryptamine, and phenylalanine treatments on glucose content. (L) Effects of tryptophan, 5‐hydroxytryptamine, and phenylalanine treatments on glucopyranose content. (M) Effects of tryptophan, 5‐hydroxytryptamine, and phenylalanine treatments on pyruvic acid content. (N) Effects of tryptophan, 5‐hydroxytryptamine, and phenylalanine treatments on citric acid content. (O) Effects of tryptophan, 5‐hydroxytryptamine, and phenylalanine treatments on acetyl‐CoA content. For (B)–(C) and (E)–(O), different letters denote significant differences (*P* < 0.05, one‐way ANOVA followed by Duncan's multiple‐range test); the absence of superscript letters denotes that no statistically significant differences were observed among the treatments; data are mean SD of three biological replicates (*n* = 3), each value averaged from four fruits.

The tryptophan synthase gene *CsTRYS* and the tryptophan decarboxylase gene *CsTDC2* were key regulatory genes in wound healing. The expression patterns of *CsTRYS* and *CsTDC2* during wound healing were examined following exogenous application of tryptophan, 5‐hydroxytryptamine, and phenylalanine. As shown in Figure 7E,F, quantitative real‐time PCR analysis revealed that treatment with these substances upregulated the expression of both *CsTRYS* and *CsTDC2* on wound healing 3 days. Compared to control, tryptophan decarboxylase (TD) activity was significantly enhanced throughout the wound healing formation period, while tryptophan synthase (TS) activity showed no significant increase (Figure 7G,H). Furthermore, among the metabolites within the amino acid metabolic pathway, endogenous levels of tyrosine and phenylalanine—structurally related aromatic amino acids—were markedly elevated following exogenous treatment with tryptophan, 5‐hydroxytryptamine, or phenylalanine (Figure 7I). Notably, endogenous tryptophan accumulation was significantly higher after treatment with tryptophan and 5‐hydroxytryptamine compared to phenylalanine treatment, whereas endogenous phenylalanine content was highest following phenylalanine supplementation. These findings indicate that exogenous application of tryptophan, 5‐hydroxytryptamine, and phenylalanine effectively enhanced gene expression levels in the amino acid metabolic pathway, increased enzymatic activities of TD, and elevated endogenous levels of tyrosine, tryptophan, and phenylalanine.

The expression of the tricarboxylic acid cycle‐related gene *CsCCL3* in wound healing was significantly upregulated following treatment with tryptophan, 5‐hydroxytryptamine, and phenylalanine in comparison to the control group (Figure 7J), with the highest induction observed after phenylalanine treatment. Glucose, glucopyranose, pyruvic acid, citric acid, and acetyl‐CoA serve as crucial intermediates in the tricarboxylic acid cycle. At the metabolite level, treatments with tryptophan, 5‐hydroxytryptamine, and phenylalanine markedly enhanced the concentrations of these metabolites (Figure 7K–O). Specifically, pyruvic acid accumulation was most pronounced following tryptophan treatment, whereas glucose, glucopyranose, citric acid, and acetyl‐CoA reached their peak contents after phenylalanine treatment. These observations demonstrate that tryptophan, 5‐hydroxytryptamine, and phenylalanine trigger activation of the tricarboxylic acid cycle and concomitant accumulation of its intermediates, which may provide metabolic substrates and energy supporting fatty acid‐derived suberin formation during wound healing responses.

### Exogenous tryptophan, 5‐hydroxytryptamine, and phenylalanine activated suberin biosynthesis of wound healing

To further investigate the relationship between the exogenous application of these compounds and wound healing, their influence on the suberin biosynthesis pathway was examined. The genes *CsKCS4*, *CsKCS11*, and *CsGPAT9* play critical roles in suberin biosynthesis in citrus fruit. Relative to the control, the expression levels of these genes showed significant upregulation following treatment with tryptophan, 5‐hydroxytryptamine, or phenylalanine (*P* < 0.05) (Figure 8A–C). Notably, phenylalanine treatment exhibited the most pronounced effect on the upregulation of *CsKCS11* and *CsGPAT9*, whereas 5‐hydroxytryptamine treatment showed the strongest induction of *CsKCS4* expression. At the metabolite level, the contents of fatty acid constituents were markedly elevated after the external application of these compounds (Figure 8D,E). In addition, the concentrations of glycerides, α,ω‐diacids, ferulic acid, and total suberin polyaliphatic (SPA) also showed significant increases after exogenous treatment (Figure 8F). These results indicate that tryptophan, 5‐hydroxytryptamine, and phenylalanine effectively promoted citrus fruit wound healing by significantly activating the suberin biosynthesis pathway.

**Figure 8:**
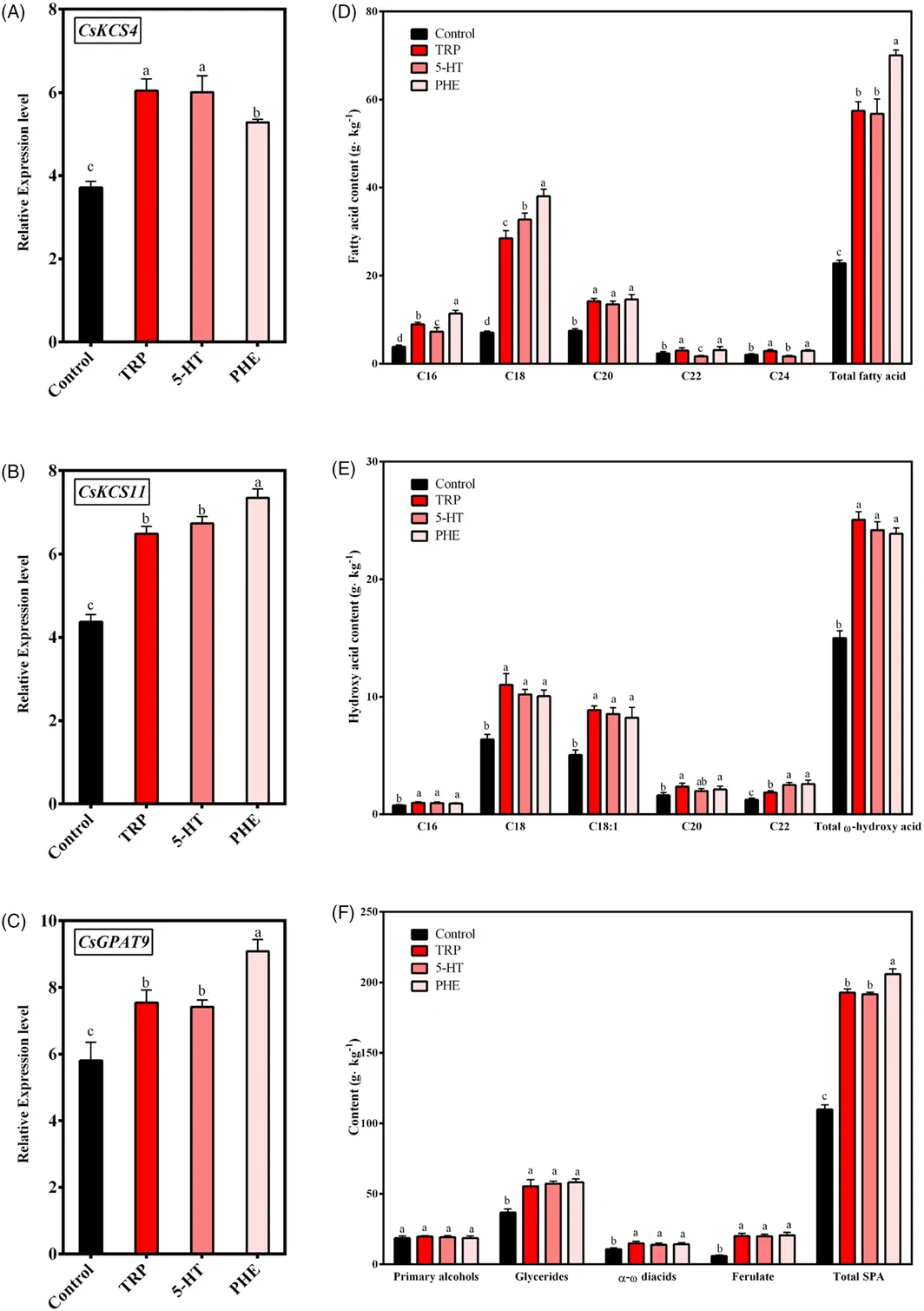
Exogenous tryptophan, 5‐hydroxytryptamine, and phenylalanine activated suberin biosynthesis of wound healing. (A) Effect of tryptophan, 5‐hydroxytryptamine, and phenylalanine treatments on the expression level of the *CsKCS4* gene in citrus fruit. (B) Effect of tryptophan, 5‐hydroxytryptamine, and phenylalanine treatments on the expression level of the *CsKCS11* gene in citrus fruit. (C) Effect of tryptophan, 5‐hydroxytryptamine, and phenylalanine treatments on the expression level of the *CsGPAT9* gene in citrus fruit. (D) Effect of tryptophan, 5‐hydroxytryptamine, and phenylalanine treatments on the content of fatty acids in citrus fruit. (E) Effect of tryptophan, 5‐hydroxytryptamine, and phenylalanine treatments on the content of hydroxy fatty acids in citrus fruit. (F) Effect of tryptophan, 5‐hydroxytryptamine, and phenylalanine treatments on the content of primary alcohols, glycerides, α,ω‐dicarboxylic acids, ferulic acid, and total suberin polyaliphatic acid (SPA) in citrus fruit. For (A–F), different letters denote significant differences (*P* < 0.05, one‐way ANOVA followed by Duncan's multiple‐range test); the absence of superscript letters denotes that no statistically significant differences were observed among the treatments; data are mean SD of three biological replicates (*n* = 3), each value averaged from four fruits.

## DISCUSSION

Wound healing represents a crucial postharvest physiological trait in citrus fruit, the effective development of which can help prevent quality deterioration and the spread of pathogenic fungal infections (Gao et al., 2024; Gao, Li, et al., 2025; Gao, Wang, et al., 2025; Huang et al., 2025; Li et al., 2026; Zhao et al., 2018). However, studies investigating the role of GATA transcription factors in wound healing are still limited. Based on our earlier transcriptomic analysis (Gao et al., 2024), a notable upregulation of the GATA‐type transcription factor CsGATA5 was detected during citrus wound healing. To gain deeper insight into the function of CsGATA5 in wound healing, its transcriptional expression pattern was analyzed, revealing a significant upregulation during the wound healing process (Figure 1A). Several studies have explored the functions of MYB, bHLH, and NAC transcription factors by analyzing their markedly increased expression levels during wound healing in various fruits, such as potato tubers (Yang et al., 2025), kiwifruit (Wei et al., 2024), and pitaya (Li et al., 2021). These findings suggest a potential association between CsGATA5 and wound healing. Furthermore, sequence alignment and phylogenetic assessment of CsGATA5 confirmed the presence of a distinct ZnF (zinc finger) protein domain and demonstrated high sequence homology with AtGATA3 from Arabidopsis (Figure 1B,C). AtGATA3 has been well established as a key regulator involved in tissue differentiation and growth in Arabidopsis (Zhang et al., 2013). Subcellular localization and transcriptional activation assays (Figure 1D–F) revealed that CsGATA5 was localized in the nucleus and, similar to BrGATA21 and CsGATA12, possesses transcriptional activation activity (Fan et al., 2024; Mou et al., 2025). Collectively, CsGATA5 may act as a transcriptional regulator in the nucleus of citrus fruit cells, exerting a transcriptional activation effect to mediate wound healing.

In order to clarify the biological function of CsGATA5 in wound healing, transient overexpression and gene silencing experiments were performed using citrus peel tissues. Suberin deposition and disease resistance have been identified as key phenotypic markers of wound healing (Gao et al., 2024; Gao, Li, et al., 2025; Huang et al., 2025). Experimental data indicated that overexpression of *CsGATA5* significantly promotes suberin accumulation and effectively suppresses disease development (Figure 2), whereas silencing of *CsGATA5* resulted in a substantial reduction in suberin content (Figure S3). Research into the involvement of transcription factors in fruit wound healing has increasingly focused on the biosynthesis of suberin. For example, the overexpression of transcription factors including AcMYC2, AcABF2, AcMYB41, and AcMYB107 has been shown to enhance lignification in kiwifruit (Wei et al., 2024; Wei, Lu, et al., 2020; Wei, Mao, et al., 2020), while overexpression of MdMYB93 promotes suberin accumulation in apple peel (Legay et al., 2016). Additionally, transient overexpression of *CsGATA5* in tobacco leaves led to enhanced suberin deposition (Figure 2F), further substantiating the hypothesis that CsGATA5 positively mediates wound healing. Suberin deposition has been recognized as critical protective mechanisms in wound healing for resisting external stress (Benatto Perino et al., 2025; Gao et al., 2024; Wei et al., 2023; Zhang et al., 2023). These findings suggest that CsGATA5 functions as a regulatory hub, potentially mediating wound healing via the modulation of suberin biosynthesis. To further investigate the regulatory functions of AcABF and AcMYB transcription factors in kiwifruit wound healing, analogous phenotypic responses were observed in tobacco (Wei, Lu, et al., 2020). Overexpression of *St* *MYB24*, *St* *MYB144*, and *St* *MYB168* in tobacco was also found to significantly enhance the accumulation of phenolic and fatty acid constituents of suberin, thereby confirming their positive regulatory roles in wound healing of potato (Han et al., 2024). These findings collectively support a potential positive involvement of CsGATA5 in regulating citrus wound healing.

Based on previous research on the vital pathways underlying citrus wound healing (Gao et al., 2024), this study revealed that CsGATA5 regulated specific pathways involved in this process. Key genes associated with amino acid metabolism were upregulated by *CsGATA5* overexpression, resulting in the accumulation of corresponding metabolites during citrus wound healing (Figure 3). GATA TFs specifically bind to the conserved WGATAR sequence motif to modulate their target genes (Schwechheimer et al., 2022). Furthermore, our analysis revealed that CsGATA5 specifically binds to and activates target genes within these three pathways, including *CsTRYS* and *CsTDC2*, as confirmed by DLR, EMSA, ChIP‐qPCR and Y1H assays (Figure 4). Research has shown that transcription factors are involved in fruit wound healing by precisely regulating suberin biosynthesis, the phenylpropanoid pathway, and jasmonic acid and abscisic acid signaling pathways. AcbZIP12 and AcABF2 transcriptionally activate the suberin biosynthesis genes *AcKCS* and *AcFHT* in response to abscisic acid during wound healing in kiwifruit (Han et al., 2019; Wei, Lu, et al., 2020). AcABF1‐1 and AcMYC2 activate jasmonic acid biosynthesis genes *AcLOX3* and *AcOPR3*, as well as phenylpropanoid pathway genes *AcPAL*, *AcC4H*, and *Ac4CL* in kiwifruit (Wei, Mao, et al., 2022; Wei, Wei, et al., 2022). StMYB168 activates *StPAL*, *StOMT*, and *St4CL* associated with phenylpropanoid pathway in potato tuber (Han et al., 2024). Meanwhile, StMYB24 and StMYB144 regulate fatty acid metabolism by activating *StLTP*, *StLACS*, and *StCYP* in potato tuber, while *StFHT* contributes to suberin assembly (Han et al., 2024). Our research uncovered novel pathways of wound healing involving key genes *CsTRYS* and *CsTDC2* in amino acid metabolism. However, *CsCYS‐S1*, although showing increased transcriptional levels following *CsGATA5* overexpression, lacked CsGATA5 recognition elements in its promoter, or indirectly activated by complex mechanisms.

To further investigate the association between target genes and wound healing, gene overexpression and silencing experiments were conducted on the outer pericarp of citrus for the target genes *CsTRYS* and *CsTDC2* (Figure 5). *CsGPAT9*, *CsKCS4*, and *CsKCS11* have previously been implicated in suberin biosynthesis, whereas *CsXTH24* has been associated with cell wall metabolism (Gao, Wang, et al., 2025). These results further confirmed the essential role of CsGATA5 in citrus wound healing, in conjunction with its downstream target genes involved in amino acid metabolism. Notably, the findings of the current study indicated that expression of *CsTRYS* and *CsTDC2* significantly promoted wound healing, particularly by enhancing the positive regulation of suberin accumulation (Figures 5, 6; Figure S5). Amino acid metabolism is widely acknowledged as a key primary metabolic pathway in plants, serving as a central mediator of downstream secondary metabolic pathways involved in growth, development, and stress resistance (Chithra et al., 2025; Heinemann & Hildebrandt, 2021; Kawade et al., 2023). Furthermore, we conducted analyses to investigate and validate the involvement of tryptophan metabolism‐related target genes *CsTRYS* and *CsTDC2* in wound healing. *CsTRYS* and *CsTDC2*, as key genes of tryptophan metabolism, exert a positive regulatory function in the formation of wound healing. In addition, the expression of *CsTRYS* and *CsTDC2* led to an increase and a decrease in tryptophan content (Figure 6A), respectively. The tryptophan metabolic pathway has been implicated in regulation of wound tissue repair through its interaction with the JA and IAA signaling pathways (Sheldrake, 2021; Sztein et al., 2002; Zhang et al., 2019; Zhou et al., 2020). Evidence suggests that transgenic tobacco lines expressing tryptophan decarboxylase promote the biosynthesis of hydroxycinnamic acid at wound sites (Guillet & De Luca, 2005). These findings indicate that tryptophan metabolism may serve as a key driver in the development of wound healing in citrus. Phenylalanine, tryptophan, and tyrosine have been reported to be ketogenic and glucogenic amino acids, which have the potential to activate glycolysis and TCA cycle (Sankar et al., 2024; Wang et al., 2019; Wang, Li, et al., 2023). Our current findings reveal that overexpression of *CsTRYS* and *CsTDC2* not only drives the accumulation of phenylalanine and tyrosine, but also promotes the significant enrichment of core metabolites in the TCA cycle (Figure 6B). This coordinated metabolic reprogramming strongly corroborates our proposed mechanistic hypothesis. In addition, the monomeric components of suberin were significantly enriched following overexpression of *CsTRYS* and *CsTDC2* (Figure 6C). Suberin biosynthesis has been shown to rely on sugar metabolism and TCA cycle to provide fatty acid precursors and energy (Wang, Zhao, et al., 2023). These findings indicated that overexpression of target genes *CsTRYS* and *CsTDC2* in tryptophan metabolism promoted the downstream biosynthesis of suberin in citrus.

This research further substantiated the strong association between amino acid metabolism and wound healing. Following treatment with tryptophan, 5‐hydroxytryptamine, and phenylalanine (Figure 7), pronounced suberin deposition was detected on 3d of wound healing, which aligns with the time frame linked to improved healing outcomes in prior research. The accelerated formation of suberin following application of these compounds could establish an effective physical barrier against pathogen invasion at an earlier stage, highlighting their potential for practical application in the citrus industry (Wei et al., 2024). Tryptophan, 5‐hydroxytryptamine, and phenylalanine significantly enhanced the expression of *CsTRYS* and *CsTDC2*, increased the activity of TD, and elevated the endogenous levels of tyrosine, tryptophan, and phenylalanine (Figure 7). The mechanism underlying the activation of endogenous amino acid metabolic pathways by exogenous amino acid treatment could be elucidated at multiple levels. First, substrate supplementation and precursor provision played a critical role. Evidence indicated that exogenously supplied amino acids are readily taken up by fruit and vegetable cells and served as direct precursors for synthesis of endogenous amino acids. For instance, glutamate, a key intermediate in nitrogen metabolism, can be transformed into α‐ketoglutarate to enter the TCA cycle or converted into other amino acids such as alanine and aspartate via transamination reactions (Xiong et al., 2021). Exogenous tryptophan, 5‐hydroxytryptamine, and phenylalanine treatments significantly upregulated *CsTRYS* transcription but did not cause a significant increase in TS enzyme activity. This discrepancy between elevated *CsTRYS* transcript and unchanged TS enzyme activity stems from allosteric feedback inhibition coupled with post‐transcriptional regulation (Luo & Di, 2023; Sato et al., 2022; Xue et al., 2026). As the rate‐limiting enzyme in tryptophan biosynthesis, TS is directly suppressed by accumulated endogenous tryptophan. Post‐transcriptional control further uncouples mRNA abundance from functional active enzyme yield, offsetting the effect of transcriptional upregulation. Notably, tryptophan and phenylalanine might also contribute to TCA cycle intermediates and enhance endogenous pools of tyrosine, tryptophan, and phenylalanine through similar metabolic conversions (Figure 7). Second, modulation of enzyme activities and induction of gene expression might be involved (Lee et al., 2024). Exogenous amino acids could influence allosteric regulation of key metabolic enzymes and regulate gene expression through signaling pathways associated with nutrient sensing, including the TOR and SnRK1 pathways (Coronel et al., 2025; Heinemann & Hildebrandt, 2021). For example, elevated amino acid availability activated TOR kinase, which in turn promoted the transcription of ribosomal biogenesis genes, thereby accelerating protein synthesis. Furthermore, the activation of amino acid metabolic pathways following exogenous application of tryptophan, 5‐hydroxytryptamine, and phenylalanine provided critical precursors for suberin biosynthesis (Figure 8). Nevertheless, since stable isotope ^13^C‐labelling experiments were not feasible in our experimental system, direct carbon flux from tryptophan/5‐hydroxytryptamine toward suberin polymers cannot be definitively concluded from steady state metabolomic and genetic data alone. Two non‐mutually exclusive models could explain our observations. On one hand, tryptophan, 5‐hydroxytryptamine may act as potential metabolic precursors supplying TCA derived substrates to support suberin assembly. On the other hand, tryptophan and 5‐hydroxytryptamine are well‐recognized signaling elicitors in plant stress responses, capable of crosstalk with JA, auxin, ROS‐dependent defense cascades to reprogram downstream wound associated metabolism (Bhowal et al., 2021; Myers et al., 2023; Yu et al., 2026). Thus, they may trigger suberization via signal transduction rather than serving as bulk carbon donors. Future isotope tracing work will help to discriminate the relative contribution of metabolic substrate versus signaling functions of tryptophan and 5‐hydroxytryptamine during citrus wound healing.

In conclusion, we have identified that the transcription factor CsGATA5 positively regulates wound healing in citrus fruit (Figure 9). CsGATA5 directly triggered the expression of target genes *CsTRYS* and *CsTDC2* in tryptophan metabolism. Overexpression of the target genes *CsTRYS* and *CsTDC2* activated the TCA cycle, thereby positively regulated suberin biosynthesis. Exogenous application of tryptophan, 5‐hydroxytryptamine, and phenylalanine further indicated a correlative cascade reaction among tryptophan metabolism, the TCA cycle, and suberin biosynthesis mediated by tryptophan. This study refines the precise regulatory network underlying GATA transcription factor‐mediated fruit wound healing and provides critical theoretical support for the application of exogenous amino acids as a safe and effective strategy to improve wound healing in fruits.

**Figure 9:**
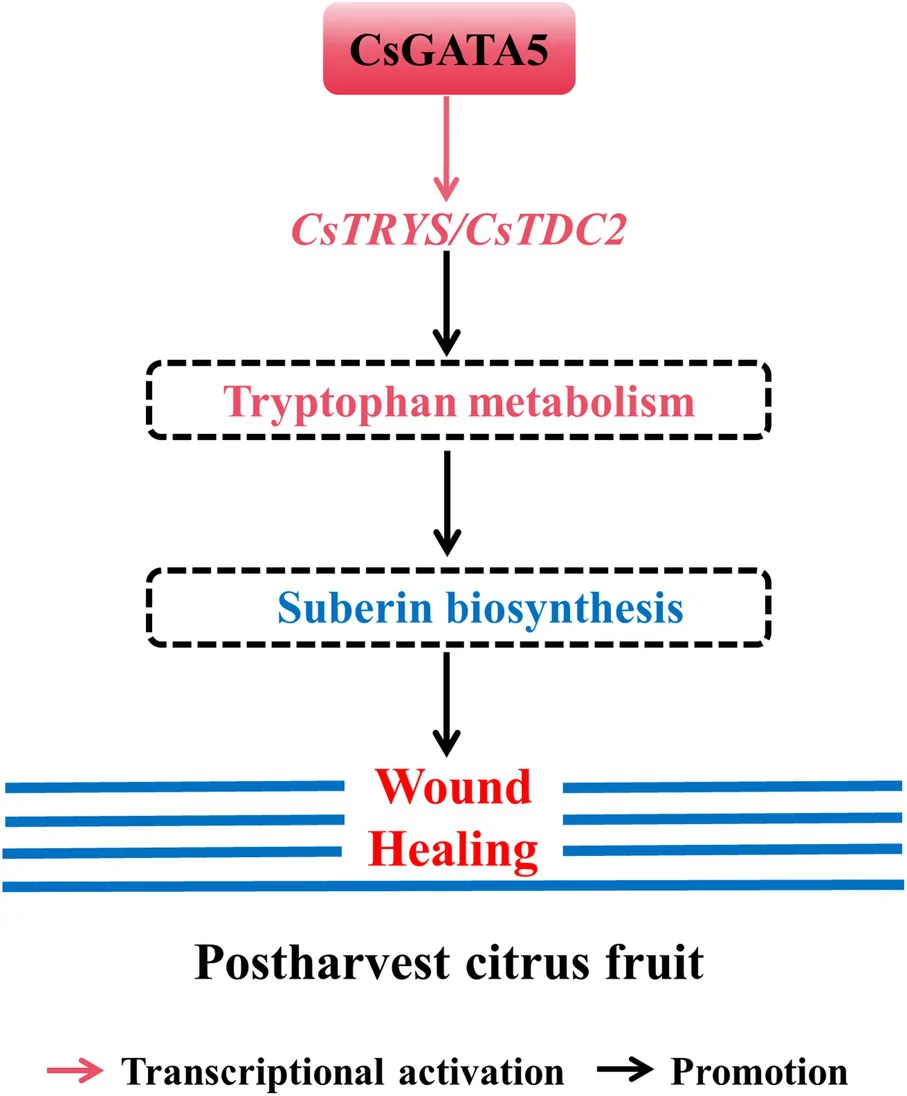
Schematic representation of the mechanism of CsGATA5‐mediated regulation in wound healing of citrus fruit.

## MATERIALS AND METHODS

### Fruit materials

The experimental procedure employed citrus fruits (*Citrus sinensis* L. Osbeck cv. Jincheng 447#) that were consistent in size and shape and free from observable symptoms of physical damage or infection. First, the fruits were immersed in a 2% sodium hypochlorite solution, thoroughly rinsed with sterile water, and then air‐dried. Subsequently, surface sterilization was performed using 75% ethanol.

### Bioinformatics identification and phylogenetic analysis of CsGATA5

The bioinformatics identification and phylogenetic analysis of CsGATA5 (Cs_ont_8g020340) were performed by DNAMAN6 and MEGA10 software following the method of Li et al. (2026).

### Subcellular localization analysis

The *CsGATA5* gene was cloned into the pCAMBIA2300‐GFP vector to examine subcellular localization according to Gao, Wang, et al. (2025). In brief, the pCAMBIA2300‐GFP‐CsGATA5 plasmid was introduced into *A. tumefaciens* strain EHA105, which was subsequently suspended in an infiltration solution composed of 10 mM MgCl_2_, 10 mM MES, and 0.1 mM Acetosyringone. The strain suspension was infiltrated into the abaxial surface of tobacco through minor punctures to achieve transient expression. GFP fluorescence in the leaf tissues was visualized using a Zeiss LSM780 confocal microscope (Carl Zeiss, Germany) after 2–3 days of incubation.

### Transcriptional activity analysis

The transcriptional activation potential of CsGATA5 was assessed employing two methods: the GAL4‐based yeast two‐hybrid system and the dual‐luciferase reporter assay, as described by Gao, Wang, et al. (2025).

### Gene expression analysis

Total RNA was extracted from citrus peel samples collected at different time points after wound healing treatment and reverse transcribed using the method of Huang et al. (2025). RT‐qPCR analysis was conducted using the PerfectStart® Uni RT&qPCR Kit (TransGen Biotech, Beijing) on a high‐throughput real‐time PCR platform. Comprehensive details for each gene, along with the associated primer sequences, are available in Tables S1 and S2 of the Supporting Information.

### Transient overexpression in citrus pericarp

The *CsGATA5*, *CsTRYS*, and *CsTDC2* genes were respectively cloned into the pCAMBIA2300 vector and subsequently transformed into the EHA105 strain. The fruit epidermis was punctured at the equatorial region using a puncher that had been surface‐sterilized with 75% ethanol, creating wounds approximately 3 mm in both diameter and depth. The empty pCAMBIA2300 plasmid served as the control in the overexpression (OE) experiment. Resuspended solutions containing *CsGATA5*, *CsTRYS*, and *CsTDC2* were injected into the wound sites in citrus pericarp. For the 0‐day time point, fruits underwent the same artificial wounding procedure and were inoculated with the overexpression suspension. Tissue samples from the wound‐adjacent areas were collected at 0 and 3 days during the healing process, under controlled conditions of 25°C and 85–90% relative humidity.

### Transient overexpression of *CsGATA5* in *Nicotiana benthamiana* leaves

The CDS of *CsGATA5* gene was inserted into pCAMBIA2300 vector under the control of the CaMV 35S promoter. The final recombinant plasmid was introduced into EHA105 strain, which was then delivered into plant leaves using a syringe infiltration method. Leaf samples were collected 3 day post‐infiltration to observe suberin deposition.

### Transient gene silencing in citrus pericarp

The 200–300 bp specific DNA fragments of *CsGATA5*, *CsTRYS*, and *CsTDC2* were respectively inserted into the pTRV2 vector to generate the silencing constructs pTRV2‐*CsGATA5*, pTRV2‐*CsTRYS*, and pTRV2*‐CsTDC2*. The empty pTRV2 vector was used as the negative control. All vectors (pTRV1, empty pTRV2, and three recombinant pTRV2 constructs) were individually transformed into EHA105. Single positive colonies were inoculated into LB liquid medium supplemented with 10 mM MES (pH 5.6), 200 μM acetosyringone, and corresponding selective antibiotics, and incubated at 28°C with shaking at 200 rpm for 16–18 h. Bacterial cells were harvested by centrifugation, resuspended in infiltration buffer (10 mM MgCl_2_, 10 mM MES, 200 μM acetosyringone, pH 5.6), and adjusted to a final OD_600_ of 0.8–1.0. The resuspended *Agrobacterium* cultures were then incubated at 28°C in darkness for 3 h to activate the virulence system before infiltration. For each gene silencing treatment, the pTRV1 suspension and the corresponding recombinant pTRV2 suspension were mixed at a volume ratio of 1:1. For the control group, pTRV1 and empty pTRV2 suspensions were mixed in the same 1:1 ratio. The mixed bacterial solutions were injected into the wound sites on citrus pericarp using a 1 mL needleless syringe. At 3 days after infiltration, pericarp tissues at the injection sites were collected for subsequent biochemical and molecular analyses.

### Exogenous application of tryptophan, 5‐hydroxytryptamine, and phenylalanine in citrus fruit

Cylindrical artificial wounds (3 mm in both diameter and depth) were created in the equatorial region of citrus fruit. Four treatment groups were established: (i) tryptophan (TRP) treatment (1 × 10^−4^ M, 10 μL); (ii) 5‐hydroxytryptamine (5‐HT) treatment (equimolar concentration, same volume); (iii) phenylalanine (PHE) treatment (equimolar concentration, same volume); and (iv) blank control group (injection of an equivalent volume of sterile water). Following natural air‐drying of the wound sites, each fruit was individually sealed and incubated under controlled conditions (25°C, 85–90% relative humidity) to facilitate wound healing. On 3 days of wound healing, a sterile punch was used to collect periwound peel tissue samples with a diameter of 5 mm. Collected samples were immediately frozen and stored at −80 C for subsequent biochemical and molecular analyses.

### Disease statistics of citrus fruit

The spore suspension of *Penicillium digitatum* was inoculated at a concentration of 1 × 10^8^ spores L^−1^, with a volume of 1 × 10^−5^ L applied to the wound area. Beginning on post‐inoculation 3 days, the disease incidence and lesion diameter were observed and recorded.

### Suberin microscope observation

Thin sections of tissue undergoing wound healing from citrus and tobacco, measuring approximately 0.2 mm in thickness and 5 mm in length, were precisely excised. Subsequently, the tissue sections were stained with toluidine blue and neutral red solution as follows: the approach described by Gao et al. (2024). Suberin deposition was visualized using an epifluorescence microscope (Eclipse TS100, Nikon, Japan).

### Analysis of amino acid content

Samples were precisely weighed (1 × 10^−3^ kg) and homogenized with 6% sulfosalicylic acid. After homogenization, the mixture was subjected to centrifugation at 12000 × ***g*** for 10 min. A 2 × 10^−5^ L aliquot of the supernatant was filtered through a 0.22 μm membrane. Amino acid content was analyzed using a Hitachi L‐8900 amino acid analyzer.

### Analysis of suberin monomers contents

The levels of suberin constituents were evaluated employing the approach described by Gao, Li, et al. (2025). Briefly, 1 mg lyophilized sample was incubated in a solution containing 2% cellulase and 2% pectinase at 40°C for 12 h. The precipitate was collected by centrifugation at 4°C and dried. The dried residue was ultrasonicated (40 MHz) in 1 mL chloroform‐methanol mixture (1:1, v/v) for 10 min, followed by another centrifugation. The resultant pellet was dried for subsequent depolymerization in 2 mL BF₃/methanol solution at 70°C for 16 h. The reaction was quenched with saturated sodium bicarbonate solution. After spiking with methyl heptadecanoate (internal standard), the mixture was extracted three times with chloroform. The organic phase was dehydrated over anhydrous sodium sulfate and concentrated under nitrogen flow. For derivatization, 100 μL BSTFA and 100 μL pyridine were added, and the mixture was heated at 70°C for 40 min. After nitrogen evaporation, the product was redissolved in 500 μL chloroform and analyzed on a GCMS‐QP2010 Plus instrument. The injector temperature was 260°C, and helium was used as carrier gas at 1 mL min^−1^. A 1 μL aliquot of sample was injected. The oven temperature program was set as follows: initial hold at 80°C for 5 min, ramped to 180°C at 3 °C min^−1^ and held for 5 min; further increased to 270 C at 6 C min^−1^ and kept for 20 min. Electron ionization was performed at 70 eV with ion‐source temperature of 230°C. Mass spectra were recorded over the m/z range of 30–550.

### Analysis of the contents of tricarboxylic acid cycle metabolites

The contents of tricarboxylic acid cycle metabolites were examined using the approach described by Gao, Li, et al. (2025) and measured by GCMS (GCMS‐QP2010, Shimadzu, Japan). In brief, 100 mg freeze‐dried sample was mixed with 1.4 mL methanol and vortexed at 950 rpm for 15 min at 70 C. The mixture was centrifuged at 10000 rpm for 10 min, and the supernatant was collected. Distilled water (1.5 mL) and chloroform (750 μL) were added to the supernatant, followed by centrifugation at 5000 rpm for 10 min. An aliquot (100 μL) of the upper aqueous phase was transferred, and 100 μL ribitol solution (0.2 kg/L) was added as internal standard. The mixture was dried in a vacuum concentrator at room temperature for 3 h. The dried residue was resuspended in 60 μL pyridine containing methoxyamine hydrochloride (20 mg/mL) and vortexed at 37 C for 90 min. Subsequently, 40 μL BSTFA reagent with 1% trimethylchlorosilane (TMCS) was added, and the sample was further vortexed at 37 C for 30 min. A 1 μL aliquot was injected into the GCMS‐QP2010 Plus instrument with a split ratio of 10:1. The injector temperature was 250 C, and helium served as carrier gas at a constant flow of 1 mL min^−1^. The oven temperature program was as follows: initial hold at 100 C for 1 min; increased to 184°C at 3°C min^−1^; ramped to 190°C at 0.5°C min^−1^ and held for 1 min; raised to 250 C at 10°C min^−1^ and held for 1 min; increased to 280 C at 5 C min^−1^, with a final hold at 280 C for 3 min. Electron ionization was performed at 70 eV, and the ion‐source temperature was set to 230 C. Mass spectra were acquired over the m/z range of 30–550.

### Dual‐luciferase reporter (DLR) assay

Wild‐type promoters harboring the WGATAR cis‐acting element of the *CsTRYS* and *CsTDC2* genes were inserted into the pGreenII 0800‐LUC vector to generate reporter gene constructs. Additionally, Shenggong Biotechnology Engineering (Shanghai) Co., Ltd. was engaged to synthesize mutant promoter variants of the *CsTRYS* and *CsTDC2* genes, in which the native WGATAR motif was replaced with the mutant sequence TTTTTT. The resulting constructs were subsequently used as templates for cloning into the pGreenII 0800‐LUC reporter vector. CsGATA5 was inserted into the pBD and pCAMBIA2300 vectors to create effectors. Following co‐incubation for 2 h with an EHA105 suspension harboring both effector and reporter plasmids, the mixture was used for transient transformation of tobacco leaves. The LUC/REN ratios were measured 48 h after infiltration using a DLR Assay Kit (11402ES, Yeasen, China).

### Electrophoretic mobility shift assay (EMSA)

The sequenced CsGATA5 CDS was inserted into a His‐tagged pCold vector and introduced into *E. coli BL21* cells. Expression of the fusion protein was initiated by adding 1 mM IPTG and incubating at 16 C overnight. The target protein was isolated using Ni resin and eluted with buffers containing imidazole. Wild‐type, cold, and mutant probes were designed based on bioinformatic analysis of the promoter sequence and predicted cis‐acting regulatory elements of *CsTRYS* and *CsTDC2* genes. The wild‐type and mutant probes were biotinylated using an EMSA Probe Biotin Labeling Kit (Beyotime, China). Protein‐DNA‐binding reactions were performed with an EMSA/Gel‐Shift Kit (Beyotime, China), and complexes were resolved by native PAGE. The shifted bands were visualized using a ChemiScope imaging system (Clinx Science Instruments).

### Chromatin immunoprecipitation (ChIP)‐qPCR

ChIP‐qPCR analysis was carried out following the method of Liu et al. (2024). Based on the computationally predicted binding site of CsGATA5 within the promoter regions of *CsTRYS* and *CsTDC2*, qPCR primers were designed (Table S2) to allow the amplification products to contain this WGATAR, with a length of 100–200 bp. The chromatin was subjected to immunoprecipitation with an anti‐CsGATA5 antibody, using pre‐immune IgG as a negative control. ChIP/Input‐DNA were employed as templates to prepare the qPCR system with 2 × Universal SYBR qPCR Mix (Blue) (Tsingke, China), and the qPCR reaction program was executed on the Archimed X4 qPCR instrument (ROCGENE, China) with three biological replicates per sample.

### Yeast one‐hybrid (Y1H) assay

A 100‐bp DNA sequence adjacent to the target gene's binding site was inserted into the pHIS2 vector. CsGATA5 was cloned into the pGADT7 vector. Two combinations were constructed: an experimental group (pHIS2‐bait + pGADT7‐CsGATA5) and a self‐activation control group (pHIS2‐bait + empty pGADT7). Both constructs were simultaneously introduced into Y187 yeast cells and grown in SD/‐Leu/‐Trp medium. Positive colonies were then selected, and serial dilutions of the cultures were spotted onto SD/‐His/‐Leu/‐Trp plates with varying concentrations of 3‐AT. The minimal 3‐AT concentration that suppressed self‐activation was used to assess interactions. Binding activity was confirmed by yeast growth on selective medium.

### Enzyme activity assays

Tissue samples were homogenized in liquid nitrogen and extracted with ice‐cold specific buffers to obtain crude enzyme extracts. All enzymatic reactions were terminated by adding trichloroacetic acid, followed by centrifugation. Absorbance was measured using a microplate reader.

Tryptophan synthase (TS): Extracted with Tris–HCl buffer (pH 7.5, containing 1 mM DTT, 0.1 mM PLP, 1 mM EDTA). Incubated with indole‐3‐glycerol phosphate and L‐serine at 37 C for 30 min. Product was quantified at 450 nm using a resorcinol reagent.

Tryptophan decarboxylase (TD): Homogenized in Tris–HCl buffer (pH 8.0, with 1 mM DTT, 0.1 mM PLP). Reacted with 2.5 mM L‐tryptophan at 37 C for 30 min. Derivative was measured at 570 nm after phenylhydrazine treatment.

### Antifungal activity assay *in vitro*

The TRP, 5‐HT, and PHE were dissolved in sterile distilled water and added to PDA medium to a final concentration of 1 × 10^−4^ M, consistent with the concentration used for *in vivo* fruit treatments. PDA plates amended with an equal volume of sterile distilled water served as the blank control. A suspension of *Penicillium digitatum* at a concentration of 1 × 10^5^ CFU/mL was spot‐inoculated at the center of each PDA plate. All plates were incubated at 25 °C in the dark. The mycelial colony diameter of each treatment was measured at 2‐, 3‐, and 4‐day post‐incubation. Each treatment was performed with three biological replicates.

### Statistical analysis

All experiments were conducted with at least three independent biological replicates. Data are expressed as mean ± standard deviation (SD). Data were analyzed by SPSS Statistics 22 (SPSS, USA) and one‐way ANOVA followed by Duncan's test for multiple comparisons. Different letters denote significant differences (*P* < 0.05). Asterisks indicate statistical significance using Student's *t*‐test; *P < 0.05; **P < 0.01. Graphs were generated using Prism 6.0 (GraphPad, USA).

## Author Contributions

Xiaoquan Gao performed the experiments, analyzed the data, and drafted the original manuscript. Xiaojiao Li and Jialin Chen performed experimental validation. Wenjun Wang refined the logical structure of this manuscript. Ou Chen assisted in revising the language of the manuscript. Lanhua Yi was responsible for funding acquisition and project administration. Kaifang Zeng designed the experiments and revised the manuscript in detail.

## Conflict of Interest

The authors declare no competing financial interests or personal relationships that could have appeared to influence the work reported in this study.

## Disclosure of AI Use

DeepSeek was used as an auxiliary tool in the final polishing phase of manuscript preparation, for cross‐checking textual logical flow and structural completeness, and refining academic English for enhanced clarity and fluency. All AI‐modified content was rigorously reviewed and validated by all co‐authors. The experimental data, scientific findings, and core conclusions are entirely original to this work, and the authors assume full responsibility for the accuracy and integrity of all manuscript content.

## Supporting information


**Table S1.** Key gene information for wound healing of citrus fruit in this study.
**Table S2.** Primer sequences used in this study.
**Figure S1.** Overexpression of *CsGATA5* promoted the accumulation of suberin monomer components in citrus fruit. Different letters denote significant differences (*P* < 0.05, one‐way ANOVA followed by Duncan's multiple‐range test); Data are mean SD of three biological replicates (*n* = 3), each value averaged from three fruits.
**Figure S2.** Overexpression of *CsGATA5* promoted suberin deposition in tobacco leaves. (A) RT‐qPCR was conducted to assess the expression levels of CsGATA5 following its overexpression in tobacco leaves; (B) Fluorescence observation of suberin accumulation in tobacco leaves following *CsGATA5* overexpression under 4 × magnification. For A, asterisks denote significant differences (**P < 0.01, Student's *t*‐test); Data are mean SD of three biological replicates (*n* = 3), each value averaged from three *N. benthamiana* leaves.
**Figure S3.** Suppression of *CsGATA5* inhibited the suberin biosynthesis in citrus fruit. (A) RT‐qPCR was conducted to assess the expression levels of CsGATA5 following its gene silencing in citrus fruit; (B) Suberin deposition was examined using fluorescence imaging in citrus fruit following gene silencing of CsGATA5; (C) The contents of suberin monomer components were quantified in citrus fruit following gene silencing of CsGATA5. For (A) and (C), asterisks denote significant differences (*P < 0.05, **P < 0.01, Student's *t*‐test); Data are mean SD of three biological replicates (*n* = 3), each value averaged from three fruits.
**Figure S4.** Overexpression of *CsGATA5* activated the accumulation of TCA cycle intermediates. (A–F) respectively denote the following intermediate metabolites of the TCA cycle: fructose, glucose, glucopyranose, pyruvate, acetyl‐CoA and citric acid. For (A–F), different letters denote significant differences (*P* < 0.05, one‐way ANOVA followed by Duncan's multiple‐range test); Data are mean SD of three biological replicates (*n* = 3), each value averaged from three fruits.
**Figure S5.** Suppression of target genes *CsTRYS* and *CsTDC2* inhibited suberin biosynthesis in citrus fruit. (A) RT‐qPCR was conducted to assess the expression levels of *CsTRYS* following its gene silencing in citrus fruit; (B) RT‐qPCR was conducted to assess the expression levels of *CsTDC2* following its gene silencing in citrus fruit; (C) Suberin deposition was examined using fluorescence imaging in citrus fruit following gene silencing of *CsTRYS* and *CsTDC2*; (D) The contents of fatty acids of suberin monomer components were quantified in citrus fruit following gene silencing of target genes *CsTRYS* and *CsTDC2*; (E) The contents of hydroxy acids of suberin monomer components were quantified in citrus fruit following gene silencing of target genes *CsTRYS* and *CsTDC2*; (F) The contents of other suberin monomer components were quantified in citrus fruit following gene silencing of target genes *CsTRYS* and *CsTDC2*. For (A) and (B), asterisks denote significant differences (**P < 0.01, Student's *t*‐test); For (D), (E), and (F), different letters denote significant differences (*P* < 0.05, one‐way ANOVA followed by Duncan's multiple‐range test); Data are mean SD of three biological replicates (*n* = 3), each value averaged from three fruits.
**Figure S6.** The effects of exogenous tryptophan, 5‐hydroxytryptamine and phenylalanine on the colony diameter of *Penicillium digitatum*. (A) Changes in colony diameter of *Penicillium digitatum* following *in vitro* inoculation with exogenous tryptophan, 5‐hydroxytryptamine, and phenylalanine over 2–4 days. (B) Photographs of *Penicillium digitatum* colonies on plates collected on day 4 post *in vitro* inoculation with exogenous tryptophan, 5‐hydroxytryptamine, and phenylalanine. For (A), identical superscript letters indicate the absence of statistically significant differences (*P* > 0.05, one‐way ANOVA followed by Duncan's multiple‐range test); Data are mean SD of three biological replicates (*n* = 3), each value averaged from three plates.

## Data Availability

All relevant data are available within the manuscript and its supplementary data.
