# Supplementary material for: CsGATA5‐CsTRYS/CsTDC2 module coordinates tryptophan‐mediated wound healing in postharvest citrus fruit
Source: Plant J. 2026 Oct 16;128(1):e71170. doi: 10.1111/tpj.71170 (PMC13673399; doi:10.1111/tpj.71170)
Supplement: Supplementary file 1 [file TPJ-128-0-s001.docx]

**Supporting Data**

**Table S1. Key gene information for wound healing of citrus fruit in this study.**

**Table S2. Primer sequences used in this study.**

**Figure S1. Overexpression of *CsGATA5* promoted the accumulation of suberin monomer components in citrus fruit.** Different letters denote significant differences (*P* < 0.05, one-way ANOVA followed by Duncan’s multiple-range test); Data are mean SD of three biological replicates (n = 3), each value averaged from three fruits.

**Figure S2. Overexpression of *CsGATA5* promoted suberin deposition in tobacco leaves.** A. RT-qPCR was conducted to assess the expression levels of CsGATA5 following its overexpression in tobacco leaves; B. Fluorescence observation of suberin accumulation in tobacco leaves following *CsGATA5* overexpression under 4 × magnification. For A , asterisks denote significant differences (***P* < 0.01, Student’s t test); Data are mean SD of three biological replicates (n = 3), each value averaged from three *N. benthamiana* leaves.

**Figure S3. Suppression of *CsGATA5* inhibited the suberin biosynthesis in citrus fruit.** A. RT-qPCR was conducted to assess the expression levels of CsGATA5 following its gene silencing in citrus fruit; B. Suberin deposition was examined using fluorescence imaging in citrus fruit following gene silencing of CsGATA5; C. The contents of suberin monomer components were quantified in citrus fruit following gene silencing of CsGATA5. For A and C, asterisks denote significant differences (**P* < 0.05, ***P* < 0.01, Student’s t test); Data are mean SD of three biological replicates (n = 3), each value averaged from three fruits.

**Figure S4. Overexpression of *CsGATA5* activated the accumulation of TCA cycle intermediates.** A-F respectively denote the following intermediate metabolites of the TCA cycle: fructose, glucose, glucopyranose, pyruvate, acetyl-CoA and citric acid. For A-F, different letters denote significant differences (*P* < 0.05, one-way ANOVA followed by Duncan’s multiple-range test); Data are mean SD of three biological replicates (n = 3), each value averaged from three fruits.

**Figure S5. Suppression of target genes *CsTRYS* and *CsTDC2* inhibited suberin biosynthesis in citrus fruit.** A. RT-qPCR was conducted to assess the expression levels of *CsTRYS* following its gene silencing in citrus fruit; B. RT-qPCR was conducted to assess the expression levels of *CsTDC2* following its gene silencing in citrus fruit; C. Suberin deposition was examined using fluorescence imaging in citrus fruit following gene silencing of *CsTRYS* and *CsTDC2*; D. The contents of fatty acids of suberin monomer components were quantified in citrus fruit following gene silencing of target genes *CsTRYS* and *CsTDC2*; E. The contents of hydroxy acids of suberin monomer components were quantified in citrus fruit following gene silencing of target genes *CsTRYS* and *CsTDC2*; F. The contents of other suberin monomer components were quantified in citrus fruit following gene silencing of target genes *CsTRYS* and *CsTDC2*. For A and B, asterisks denote significant differences (***P* < 0.01, Student’s t test); For D, E, and F, different letters denote significant differences (*P* < 0.05, one-way ANOVA followed by Duncan’s multiple-range test); Data are mean SD of three biological replicates (n = 3), each value averaged from three fruits.

**Figure S6. The effects of exogenous tryptophan, 5-hydroxytryptamine and phenylalanine on the colony diameter of *Penicillium digitatum*.** A. Changes in colony diameter of *Penicillium digitatum* following *in vitro* inoculation with exogenous tryptophan, 5-hydroxytryptamine, and phenylalanine over 2–4 days. B. Photographs of *Penicillium digitatum* colonies on plates collected on day 4 post *in vitro* inoculation with exogenous tryptophan, 5-hydroxytryptamine, and phenylalanine. For A, identical superscript letters indicate the absence of statistically significant differences (*P* > 0.05, one-way ANOVA followed by Duncan’s multiple-range test); Data are mean SD of three biological replicates (n = 3), each value averaged from three plates.

**Table S1**

| Pathway | Gene ID (Citrus sinensis v3.0) | Description | Gene name | Significant/regulate | Log_2_FC |
| --- | --- | --- | --- | --- | --- |
| Transcription factor | Cs_ont_8g020340 | Transcription factor GATA5 | *CsGATA5* | Yes/Up | 1.344843565 |
| Amino acid metabolism | Cs_ont_4g024260 | Arogenate dehydrogenase 1 | *CsAD1* | Yes/Up | 1.201835507 |
|  | Cs_ont_3g027930 | Tryptophan synthase | *CsTRYS* | Yes/Up | 1.85951953691 |
|  | Cs_ont_2g020700 | Tryptophan decarboxylase TDC2-like | *CsTDC2* | Yes/Up | 1.826454950 |
|  | Cs_ont_5g003370 | Cysteine synthase 1 | *CsCYS-S1* | Yes/Up | 1.888543240 |
| Carbohydrate metabolism | Cs_ont_9g001740 | Acetyl-coa carboxylase ligase3 | *CsCCL3* | Yes/Up | 1.020854553 |
| Suberin synthesis | Cs_ont_8g021680 | 3-Ketoacyl-CoA synthase 4 | *CsKCS4* | Yes/Up | 1.086941611 |
|  | Cs_ont_4g006740 | 3-Ketoacyl-CoA synthase 11 | *CsKCS11* | Yes/Up | 1.557365902 |
|  | Cs_ont_8g028840 | Glycerol-3-phosphate acyltransferase 9 | *CsGPAT9* | Yes/Up | 1.140228279 |

**Table S2**

| Gene name | Primer sequence |
| --- | --- |
| **RT-qPCR** |  |
| *CsGATA5-For* | AGAACGAACCCGGTCTGAAC |
| *CsGATA5-Rev* | TGGTTCGTTTGGTTCTCGCT |
| *CsTRYS-For* | ATGGAAACGCAGCATGGAGA |
| *CsTRYS-Rev* | ATGCACCACTGTATCCACCG |
| *CsTDC2-For* | TTGATAGATTTGGCCGCCGT |
| *CsTDC2-Rev* | AGTGGCACGGATCAAGACAG |
| *CsCYS-S1-For* | AAGCAGCAAGTAGCAAGGGA |
| *CsCYS-S1-Rev* | AAGCAGCAAGTAGCAAGGGA |
| *CsAD1-For* | TGACCCCACTGCTGACTTTC |
| *CsAD1-Rev* | TCATCGGCTGGTTTTTGGGA |
| *CsCCL3-For* | GTGGATCTGTAGCTGTGCCA |
| *CsCCL3-Rev* | CCAGAGCCACAGCCTTGTT |
| *CsKCS4-For* | ACCAGTTGTGGCTCCATCTC |
| *CsKCS4-Rev* | AGATGAGAAGGAGGGCGGTA |
| *CsKCS11-For* | ATTCAGAAGCCAAGGGCAGG |
| *CsKCS11-Rev* | GGGTTCTTCTCCTTTGCCGA |
| *CsGPAT9-For* | TGGAACAGTCGGAAGCAGTC |
| *CsGPAT9-Rev* | CGTCACAAACAACAGCCCAG |
| **Overexpression** |  |
| *OE-CsGATA5-For* | caatttactattctagggtaccATGGAGTTTTGCATGGAAGC |
| *OE-CsGATA5-Rev* | GGTATCTAGACTGCAGTCAAAAACTCTGAACCATGTGG |
| *OE-CsTRYS-For* | caatttactattctagggtaccATGGCTGCTCTGAAAGTAACC |
| *OE-CsTRYS-Rev* | GGTATCTAGACTGCAGTCATGGCAAAGCAAATCTTAAAGAC |
| *OE-CsTDC2-For* | caatttactattctagggtaccATGGGTAGCCTTGATTCAAACAT |
| *OE-CsTDC2-Rev* | GGTATCTAGACTGCAGCTAGAAAGCGGAGCCTTTAAACA |
| **Gene Silencing** |  |
| *VIGs-CsGATA5-For* | CGCGTGAGCTCGGTACCCCATTAGCAGACCGCAAG |
| *VIGs-CsGATA5-Rev* | AGATTCTGTGAGTAAGGTTACCGAATTCGGACAACACTCAAGTTTCAGTTTC |
| *VIGs-CsTRYS-For* | CGCGTGAGCTCGGTACCGTACATCTGTTAGTGACCGAGTTC |
| *VIGs-CsTRYS-Rev* | AGATTCTGTGAGTAAGGTTACCGAATTCTGGCAAAGCAAATCTTAAAGACTT |
| *VIGs-CsTDC2-For* | CGCGTGAGCTCGGTACCTATCCGCATTCTGGTTCGG |
| *VIGs-CsTDC2-Rev* | AGATTCTGTGAGTAAGGTTACCGAATTCGAAAGCGGAGCCTTTAAACAAC |
| **Subcellular localization** |  |
| *CsGATA5-GFP-For* | ATTTACTATTCTAGGGTACCATGGAGTTTTGCATGGAAGC |
| *CsGATA5-GFP-Rev* | CCATTCTAGACTGCAGAAAACTCTGAACCATGTGG |
| **Transcriptional activation** |  |
| *CsGATA5-pGBKT-For* | aggccgaattcccggggatccATGGAGTTTTGCATGGAAGC |
| *CsGATA5-pGBKT-Rev* | ctagttatgcggccgctgcagTCAAAAACTCTGAACCATGTGG |
| *CsGATA5-pBD-For* | tcgccgaccggtaggcctATGGAGTTTTGCATGGAAGC |
| *CsGATA5-pBD-Rev* | aaccagagttaaaggcctAAAACTCTGAACCATGTGG |
| **DLR** |  |
| *CsTRYS pro-0800-For* | cactatagggcgaattggCCCCAAATCTAATTCAAGAGTCCA |
| *CsTRYS pro-0800-Rev* | ttggcgtcttccatggGATTGTATGTAAAGAGGTTATCTACTAGTTCC |
| *CsTDC2 pro-0800-For* | cactatagggcgaattggCTCTTAAGTGTAAATAGTAGAAATATGTTGG |
| *CsTDC2 pro-0800-Rev* | ttggcgtcttccatggCTTTAATATTAACGACCGATGAAAATACT |
| *CsTRYS-M pro-0800-For* | cactatagggcgaattggGGTACCGCTGAAGTCATTCT |
| *CsTRYS-M pro-0800-Rev* | ttggcgtcttccatggCTCGAGAATATTATTATTAG |
| *CsTDC2-M pro-0800-For* | cactatagggcgaattggGGTACCATATTTCATATT |
| *CsTDC2-M pro-0800-Rev* | ttggcgtcttccatggCTCGAGATTACAAATATTA |
| **Y1H** |  |
| *CsTRYS pro-pHIS2-For* | cgactcactatagggcgaattcGTCAATACGAGTAGAAAACCGTCA |
| *CsTRYS pro-pHIS2-Rev* | tcgcgaacgcgtgagctcGATTGTATGTAAAGAGGTTATCTACTAGTTCC |
| *CsTDC2 pro-pHIS2-For* | cgactcactatagggcgaattcGAGGGACTGCACAGTAATTTACC |
| *CsTDC2 pro-pHIS2-Rev* | tcgcgaacgcgtgagctcCGTCGGTTCGGTTCGA |
| **EMSA** |  |
| *CsTRYS-probe-For* | TGGGAACTAGTAGATAACCTCTTTACA |
| *CsTRYS-probe-Rev* | TGTAAAGAGGTTATCTACTAGTTCCCA |
| *CsTRYS-mutant probe-For* | TGGGAACTAGTTTTTTTCCTCTTTACA |
| *CsTRYS-mutant probe-Rev* | TGTAAAGAGGAAAAAAACTAGTTCCCA |
| *CsTDC2-probe-For* | AGTTAGTAAGATATTAGTGATAAAATGTGT |
| *CsTDC2-probe-Rev* | ACACATTTTATCACTAATATCTTACTAACT |
| *CsTDC2-mutant probe-For* | AGTTAGTAAGATATTAGTTTTTTAATGTGT |
| *CsTDC2-mutant probe-Rev* | ACACATTAAAAAACTAATATCTTACTAACT |
| **Chip-qPCR** |  |
| *CsTRYS pro-For* | TGCTGAAGTCATTCTCTTGAGG |
| *CsTRYS pro-Rev* | GAGTTACTCAGATAGACGAAGC |
| *CsTDC2 pro-For* | GGGAGTATCTTATTAATAGT |
| *CsTDC2 pro-Rev* | TTCAACAATTAATTTATCCT |

**Figure S1**

**Figure S2**

**Figure S3**

**Figure S4**

**Figure S5**

**Figure S6**
